# Integrating Genome-Wide Genetic Variations and Monocyte Expression Data Reveals *Trans*-Regulated Gene Modules in Humans

**DOI:** 10.1371/journal.pgen.1002367

**Published:** 2011-12-01

**Authors:** Maxime Rotival, Tanja Zeller, Philipp S. Wild, Seraya Maouche, Silke Szymczak, Arne Schillert, Raphaele Castagné, Arne Deiseroth, Carole Proust, Jessy Brocheton, Tiphaine Godefroy, Claire Perret, Marine Germain, Medea Eleftheriadis, Christoph R. Sinning, Renate B. Schnabel, Edith Lubos, Karl J. Lackner, Heidi Rossmann, Thomas Münzel, Augusto Rendon, Cardiogenics Consortium, Jeanette Erdmann, Panos Deloukas, Christian Hengstenberg, Patrick Diemert, Gilles Montalescot, Willem H. Ouwehand, Nilesh J. Samani, Heribert Schunkert, David-Alexandre Tregouet, Andreas Ziegler, Alison H. Goodall, François Cambien, Laurence Tiret, Stefan Blankenberg

**Affiliations:** 1 INSERM UMRS 937, Pierre and Marie Curie University (UPMC, Paris 6) and Medical School, Paris, France; 2 II. Medizinische Klinik und Poliklinik, Universitätsmedizin der Johannes-Gutenberg Universität Mainz, Mainz, Germany; 3 Medizinische Klinik II, Universität Lübeck, Lübeck, Germany; 4 Institut für Medizinische Biometrie und Statistik, Universität Lübeck, Lübeck, Germany; 5 Institut für Klinische Chemie und Laboratoriumsmedizin, Universitätsmedizin der Johannes-Gutenberg Universität Mainz, Mainz, Germany; 6 Department of Haematology, University of Cambridge and National Health Service Blood and Transplant, Cambridge, United Kingdom; 7 MRC Biostatistics Unit, Cambridge, United Kingdom; 8 Human Genetics, Wellcome Trust Sanger Institute, Hinxton, United Kingdom; 9 Klinik und Poliklinik für Innere Medizin II, Universität Regensburg, Regensburg, Germany; 10 Department of Cardiovascular Sciences, University of Leicester, Leicester, United Kingdom; 11 Leicester NIHR Biomedical Research Unit in Cardiovascular Disease, Leicester, United Kingdom; Stanford University School of Medicine, United States of America

## Abstract

One major expectation from the transcriptome in humans is to characterize the biological basis of associations identified by genome-wide association studies. So far, few *cis* expression quantitative trait loci (eQTLs) have been reliably related to disease susceptibility. *Trans*-regulating mechanisms may play a more prominent role in disease susceptibility. We analyzed 12,808 genes detected in at least 5% of circulating monocyte samples from a population-based sample of 1,490 European unrelated subjects. We applied a method of extraction of expression patterns—independent component analysis—to identify sets of co-regulated genes. These patterns were then related to 675,350 SNPs to identify major *trans*-acting regulators. We detected three genomic regions significantly associated with co-regulated gene modules. Association of these loci with multiple expression traits was replicated in Cardiogenics, an independent study in which expression profiles of monocytes were available in 758 subjects. The locus 12q13 (lead SNP rs11171739), previously identified as a type 1 diabetes locus, was associated with a pattern including two *cis* eQTLs, *RPS26* and *SUOX*, and 5 *trans* eQTLs, one of which (*MADCAM1*) is a potential candidate for mediating T1D susceptibility. The locus 12q24 (lead SNP rs653178), which has demonstrated extensive disease pleiotropy, including type 1 diabetes, hypertension, and celiac disease, was associated to a pattern strongly correlating to blood pressure level. The strongest *trans* eQTL in this pattern was *CRIP1*, a known marker of cellular proliferation in cancer. The locus 12q15 (lead SNP rs11177644) was associated with a pattern driven by two *cis* eQTLs, *LYZ* and *YEATS4*, and including 34 *trans* eQTLs, several of them tumor-related genes. This study shows that a method exploiting the structure of co-expressions among genes can help identify genomic regions involved in *trans* regulation of sets of genes and can provide clues for understanding the mechanisms linking genome-wide association loci to disease.

## Author Summary

One major expectation from the transcriptome in humans is to help characterize the biological basis of associations identified by genome-wide association studies. Here, we take advantage of recent technical and methodological advances to examine the influence of natural genetic variability on >12,000 genes expressed in the monocyte, a blood cell playing a key role in immunity-related disorders and atherosclerosis. By examining 1,490 European population-based subjects, we identify three regions of the genome reproducibly associated with specific patterns of gene expression. Two of these regions overlap genetic variants previously known to be involved in the susceptibility to type 1 diabetes, celiac disease, and hypertension. Genes whose expression is modulated by these genetic variants may act as mediators in the causal relationship linking the variability of the genome to complex disease. These findings illustrate how integration of genetic and transcriptomic data at an epidemiological scale can help decipher the genetic basis of complex diseases.

## Introduction

Owing to the development of genome-wide association studies (GWAS), the last two years have witnessed spectacular successes in the identification of new loci involved in the susceptibility to complex diseases [1]. However, most of these associations have yet to be translated into a full understanding of the genetic mechanisms that are mediating disease susceptibility. The possibility of assaying genome-wide expression (GWE) and genome-wide variability (GWV) simultaneously in large-scale studies opens new perspectives for unravelling these mechanisms [2].

Several studies on the genetics of expression have shown that a considerable number of genes are regulated by expression SNPs and that *cis* expression quantitative loci (eQTLs) largely outnumber *trans* eQTLs [3]–[8]. A reason for this imbalance might be that *trans* eQTLs are beneath the level of detection of most studies because, unlike *cis* eQTLs, they do not directly influence gene expression. Moreover, *trans* associations are more sensitive to confounding factors including technical experimental effects and stratification of the cell population [9].

Large-scale transcriptional modules, i.e. sets of genes highly co-regulated, which are thought to be involved in pathophysiological processes [10], have been described in yeast [11], [12], *Drosophila* [13], mice [14]–[16] and humans [6]. Identification of *trans*-acting SNPs affecting such transcriptional modules might enhance our understanding of the molecular mechanisms involved in pathophysiological processes. Since such *trans*-acting SNPs are expected to have pleiotropic effects on a large number of genes, each being modestly affected, their statistical mapping may be facilitated by prior recognition of subsets of co-regulated genes.

In the present study, we have analyzed 12,808 genes expressed in circulating monocytes in relation to GWV in a population-based sample of 1,490 unrelated subjects participating in the Gutenberg Health Study (GHS). We applied first a method of extraction of expression patterns – independent component analysis (ICA) [17]–[19] – in order to identify sets of co-regulated genes. These patterns were then related to 675,350 SNPs to identify major *trans*-acting regulators. We identified three genomic regions, centered on the *ERBB3*, *SH2B3* and *LYZ-YEATS4* genes respectively, that were associated to expression patterns. Connecting these results with recent GWAS findings provided potential clues for better understanding the genetic basis of complex diseases.

## Results

The study was conducted in 1,490 individuals of European origin (730 women and 760 men) aged 35 to 74 years that were recruited in the GHS, a community-based project conducted in a single centre in the region of Mainz (Germany) [20]. Monocytes were freshly isolated from peripheral blood by negative separation using a cocktail of antibodies directed against non-monocytic cells (CD2, CD3, CD8, CD19, CD56 and CD66b). GWE profiles were generated using *Illumina* Human HT12 BeadChip expression arrays, and after normalization and filtering out of genes whose expression was under the detection level in significantly more than 95% of samples and genes not well characterized, 12,808 expression traits (averaged over probes) remained for analysis (see Methods).

### Description of ICA method

The goal of ICA [18], [19] is to find hidden variables, called “independent components”, which represent underlying processes that influence gene expression. The expression of each gene is written as a linear function of these components, where the influences of different components show minimal statistical dependencies. Each component defines groups of co-induced and/or co-repressed genes. These components may be viewed as reflecting distinct biological causes influencing gene expression, such as activation of signaling pathways, binding of transcription factors, posttranscriptional regulation…

**Figure 1:**
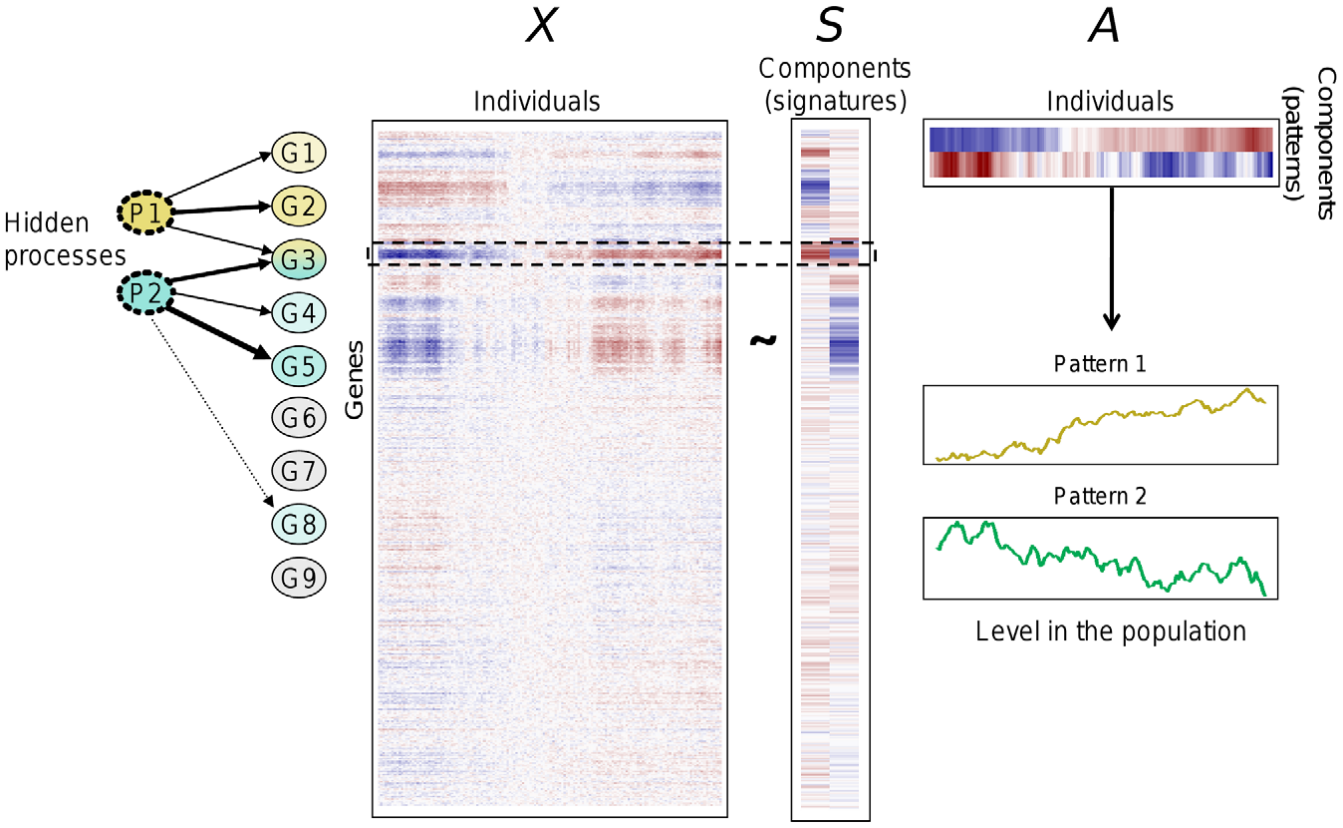
An example of the ICA method with *K* = 2 (*K*, number of independent components). Data are represented using a heat color map, from dark blue (minimum) to dark red (maximum). ICA splits the gene expression matrix *X* into a matrix product *X* = *SA*, introducing two new components (“signatures”, contained in the columns of *S*) with minimal statistical dependencies between them. These components may be viewed as reflecting hidden underlying processes influencing gene expressions (*P1* and *P2*). In the example, *P1* influences 3 genes and *P2* influences 4 genes. Gene *G3* is influenced by both processes, which is reflected by the dark red and dark blue colors in the row corresponding to *G3 i*n matrix *S*. The rows of matrix *A* represent the levels of the two components in individuals (“patterns”). The same data are shown as continuous profiles below. Individuals have been ordered to show that when levels of pattern 1 increase, levels of pattern 2 decrease, resulting in a negative correlation between the two patterns.

We consider an expression data matrix *X* whose rows correspond to genes and columns to individuals. The ICA model splits the matrix into a matrix product *X*∼*SA* (see Figure 1), subject to the condition that the statistical dependence between the *K* columns of *S* be minimized. The expression level of gene *i* in individual *j* is
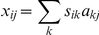
where *s_ik_* is the contribution of component *k* on gene expression *i* and *a_kj_* is the level of “activation” of that component in individual *j*. Note that the components can be interpreted in a dual view. First, each column of *S* is a vector of the linear contributions of the component on each gene expression which can be interpreted as the “signature” of the underlying biological process. To minimize the dependence between the columns of *S*, ICA identifies components that exhibit approximately sparse signatures, showing an increased proportion of contributions close to zero. Each component can then be characterized by a set of genes for which its contributions are “significantly” different from zero (see the definition of modules below). Importantly, different components can be characterized by overlapping sets of genes. For this reason, ICA is likely to better reflect biological reality than methods that partition genes into distinct clusters.

Alternatively, each component can be characterized by its pattern of expression in individuals (rows of *A*) which reflects the level of “activation” of the underlying biological process. Pattern levels are estimated by linear combinations of gene expression levels obtained by inverting the equation *X*∼*SA*. Patterns can be correlated with each other in the population. This is an advantage of ICA over classical methods of dimensionality reduction relying on orthogonality of factors like principal component analysis (PCA) [21], [22].

In the following, we used the term of “signature” or that of “pattern” for a component according to whether it referred to columns of *S* (genes) or rows of *A* (individuals). Figure 1 shows an illustration of ICA for *K* = 2.

### Analysis workflow

**Figure 2:**
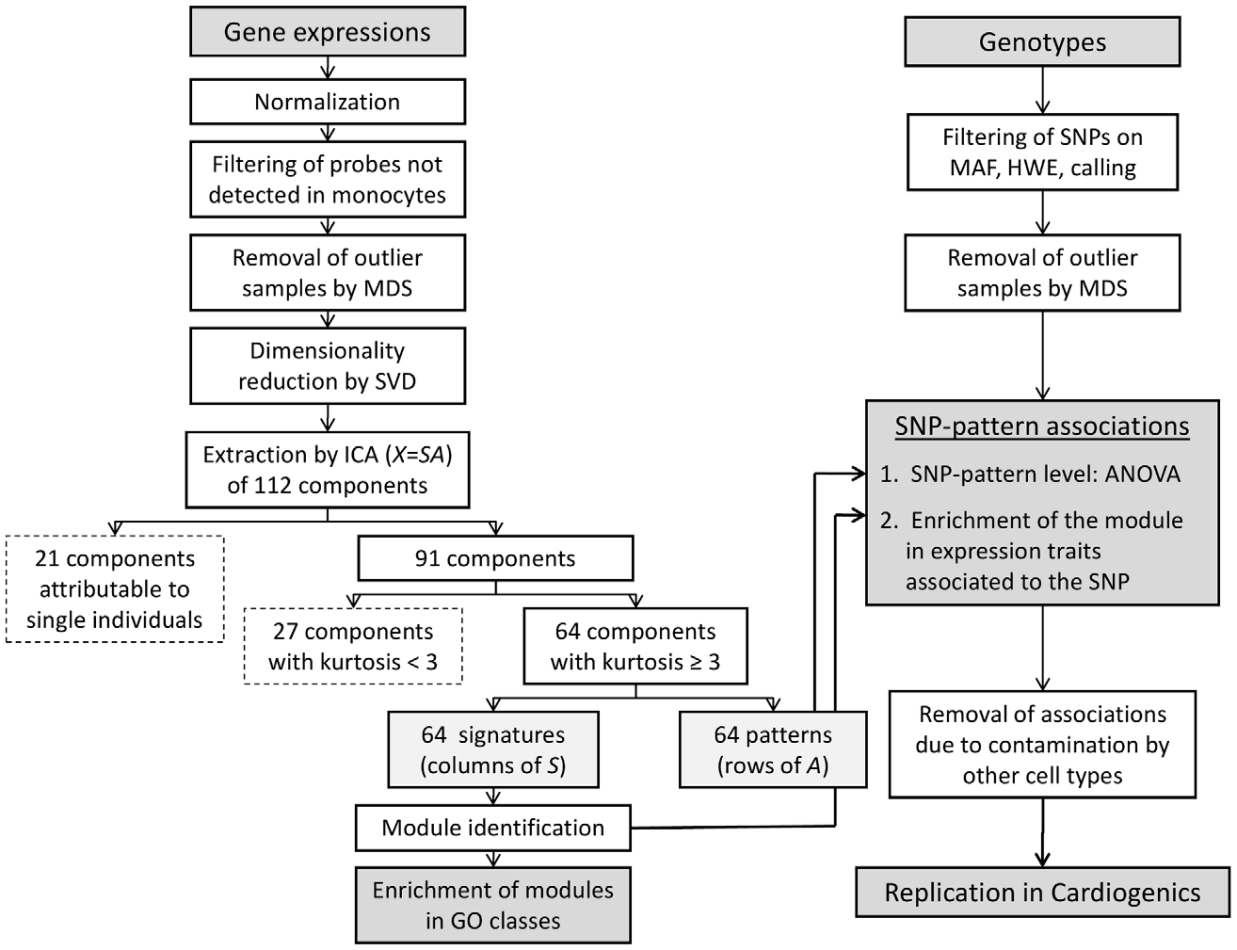
Analysis workflow. The graph shows the workflows used in parallel for expression data and genotype data. MDS: multidimensional scaling; SVD: singular value decomposition; ICA: independent component analysis; GO: gene ontology; MAF: minor allele frequency; HWE: Hardy-Weinberg equilibrium.

Figure 2 shows the analysis workflow. After normalization of raw expression data, filtering of undetected probes and removal of outlier samples by multi-dimensional scaling (MDS) analysis (Figures S1, S2, S3, S4), singular value decomposition (SVD) was used prior to ICA to reduce the dimensionality of data and determine the optimal number of components to extract by ICA [18], [19] (see Text S1). As shown by the SVD screeplot (Figure S5), 30 orthogonal components were able to capture 50% of the global variability of the transcriptome. However, as we were interested in components potentially explaining small, but meaningful, variations of the transcriptome, we extended the number of components up to the limit beyond which variability appeared mostly attributable to random noise. According to the SVD screeplot, this limit was 112 (Text S1). The FastICA algorithm was then run with this fixed number of components.

Twenty-one of the 112 components identified by ICA were characterized by a single individual who explained more than 10% of the variability of the pattern in the population. Actually, we found that most of these individuals, although not having been initially identified as outliers, were at the periphery of the main cluster of individuals obtained from the MDS analysis of expression data performed prior to ICA (Text S1, Figure S4). These 21 “individual-specific” components were no longer considered, leaving 91 components for further analysis.

### Modules of genes characterizing signatures

The fundamental principle of ICA estimation is that the columns of *S* (signatures) must be as non-gaussian as possible, typically a peaked distribution with few genes at the tails to which the signature strongly contributes, and the majority of genes in the center being weakly or not influenced [18], [19]. To determine the most non-gaussian signatures, hence the most informative components, we used the kurtosis which measures the peakedness of the distribution [18] and focused on signatures showing a kurtosis ≥3. This criterion led to the selection of 64 signatures. As explained above, the 64 signatures correspond to 64 patterns of expression in the population. Some of these patterns exhibited strong pairwise correlations (see the correlation matrix in the GHS_ICA_Modules database at http://genecanvas.ecgene.net/uploads/ForReview/).

For each of these 64 signatures, we defined the “module” as the subset of genes the most strongly influenced, i.e. genes at both extremes of the distribution. For this purpose, we used a method proposed for false discovery rate (FDR) estimation [23]. Genes associated with an FDR<10^−3^ were considered as belonging to the module characterizing the signature. The size of the modules varied from 14 to 670 genes (median 179).

### Gene Ontology enrichment analysis of modules

A Gene Ontology (GO) analysis was performed to identify modules that were associated with specific biological processes. For 42 of the 64 modules (66%), we found a significant enrichment of GO classes from genes of the module (Table S1). Over-represented biological categories included a large number of categories related to immune and inflammatory response (reponse to virus, T-cell activation, response to bacteria/fungus, cytokine activity, acute inflammatory response, humoral immune response …) and several low level biological process categories such as the nucleotide metabolic process, mRNA metabolic process, ribosome biogenesis, regulation of cell proliferation, nucleosome assembly (histone genes), and cell-cycle.

### Association between patterns and genome-wide variability (GWV)

We next investigated whether the level of expression of patterns in the population was influenced by SNPs. GWV genotyping was carried out using *Affymetrix* SNP Array 6.0. After quality control filters, 675,350 SNPs were available for testing association with the 64 patterns.

Association between patterns and SNPs was tested in a 2-step approach (Figure 2). First, we applied a filtering to select SNP-pattern associations that were significant at *P*<10^−7^ (suggestive associations). The significance threshold used in this first step was taken not too stringent in order to increase the sensitivity. The second step was aimed at discarding the SNP-pattern associations that were almost entirely explained by a single or very few genes of the module whose expression strongly correlated to the SNP. This would be the case, for example, for a SNP having a strong effect on a *cis* eQTL belonging to the module, but not associated with any other expression trait of the module. To exclude these cases of less interest for the present study, a SNP-pattern association was retained at step 2 if the corresponding module was significantly enriched in expression traits individually associated to the SNP by reference to the whole set of expression traits. Since the goal here was to detect associations not necessarily very strong but clustering within modules, a threshold of *P*<10^−5^ was taken for associations between SNP and individual expression traits (the threshold adopted for results reported in the publicly available GHS_Express database of SNP-expression associations http://genecanvas.ecgene.net/uploads/ForReview/). Enrichment of the module in significant associations was tested using a hypergeometric test with a threshold of significance of 1.15×10^−9^ (Bonferroni-corrected for 64 modules×675,350 SNPs).

This 2-step approach led to the detection of 11 patterns associated with one or several SNPs at the same locus. The proportion of variability of the pattern explained by the lead SNP at the locus varied from 1.9% to 24.8% (Table 1). Because the method of monocyte enrichment did not yield a 100% purity and even modest heterogeneity of cell content may induce artefactual correlations among expressions [24], we checked whether contamination by non-monocyte cells might affect the associations observed. For this purpose, we generated surrogate variables of contamination corresponding to each blood cell type reported in the HaemAtlas [25]. We created 7 variables corresponding to the different cell types (CD4+, CD8+, CD19+, CD56+, CD66b+, erythroblasts and megakaryocytes) by averaging in each individual his (her) levels of expression for the transcripts reported to be specific of that cell type. When re-testing the 11 SNP-pattern associations by multiple regression analysis simultaneously adjusting for the 7 contamination variables, 5 associations lost significance (Table 1). Worthy of note, the corresponding modules were enriched in GO categories relevant for the incriminated cell types (Table S1). Moreover, in several cases the best associated SNP was located in a gene highly relevant to the type of cell: the *ARHGEF3* gene, which has been reported to influence mean platelet volume [26], was involved in potential contamination by platelets; the *CD8A* gene, encoding the alpha chain of the CD8 antigen found on T cells, was involved in the level of likely contamination by T cells; the *MAP3K7* gene, a gene involved in B-cell specific immune response [27], was involved in the level of contamination by B cells. Following the same reasoning, we might anticipate a biological link between the *MAGI2* gene and potential contamination by erythroblast-derived cells (Table 1).

**Table 1:** Genome-wide association of SNPs with patterns.

Pattern	Lead SNP associated to the pattern at the locus	Chr	Position (bp)	Genes nearby (genes *cis* regulated by the SNP are underlined)	*P*-value for the SNP-pattern association^a^	Variance of the pattern explained by the SNP (R^2^)	Number of genes within the module	Number of expression traits associated to the SNP within the module^b^	*P*-value for enrichment in expression traits associated to the SNP within the module^c^	SNP-pattern association potentially due to contamination (type of cell incriminated)
33	rs2300573	1	166560874	*TBX19*	3.15 E-08	0.023	176	18	1.25 E-34	No
21	rs13023213	2	86875454	*CD8A*	7.52 E-08	0.022	292	36	3.08 E-59	Yes (T cells)
12	rs12485738	3	56840816	*ARHGEF3*	8.76 E-24	0.069	379	288	<1.0 E 250	Yes (platelets)
93	rs1344142	3	56832473	*ARHGEF3*	1.48 E-18	0.054	135	61	1.05 E-60	Yes (platelets)
48	rs13196564	6	91563760	*MAP3K7*	5.24 E-08	0.022	311	7	5.24 E-10	Yes (B cells)
66	rs2842892	6	132856076	*STX7*	9.40 E-08	0.019	137	5	1.30 E-10	No
35	rs12705417	7	77856777	*MAGI2*	1.23 E-08	0.024	395	10	6.96 E-16	Yes (erythrocytes)
7	rs1058348	10	11342351	*CUGBP2*	3.49 E-08	0.020	189	49	1.45 E-46	No
62	rs653178	12	110492139	*ATXN2, SH2B3*	2.36 E-09	0.026	62	5	5.49 E-10	No
98	rs11177644	12	68072015	*LYZ, YEATS4*	1.14 E-92	0.248	45	36	1.22 E-86	No
102	rs11171739	12	54756892	*RPS26, SUOX*	2.89 E-70	0.194	14	7	2.45 E-21	No

a *P*-values<10^−7^ were considered for SNP-pattern associations;

b *P*-values<10^−5^ were considered for associations between the SNP and expression traits within the module;

c *P*-values<1.15×10^−9^ were considered for the enrichment in expression traits associated to the SNP within the module.

For associations that were not affected by potential contamination, we checked whether they replicated in the Cardiogenics Study in which monocyte GWE profiles and GWV genotypes were available in 758 subjects (see Methods). Replication in Cardiogenics was assessed by examining the association between the lead SNP (or a proxy when it was not available) and each expression trait of the module. For three of the SNP-pattern associations (rs1058348-pattern7, rs2300573-pattern33 and rs2842892-pattern66), replication was not achieved in Cardiogenics as none of the expression traits in the module was significantly associated to the SNP. Detailed results of these associations are available in the GHS_ICA_modules database (http://genecanvas.ecgene.net/uploads/ForReview/). Worthy of note, module of pattern 33 was strongly enriched in genes involved in the immune response and largely overlapped with the recently identified rat network centered on the transcription factor IRF7, a master regulator of the type-1 interferon response [28]. For three modules, at least two expression traits were significantly associated to the SNP at a Bonferroni-corrected threshold, and a significant enrichment in genes associated with the SNP was observed at the suggestive threshold of 10^−3^.

### Association of pattern 102 with locus 12q13 involved in type 1 diabetes (T1D) susceptibility

The association between pattern 102 and rs11171739 on chromosome 12q13 (*P* = 2.9×10^−70^ for association, *P* = 2.5×10^−21^ for enrichment) is of particular interest as rs11171739 has been identified by GWAS as a marker for T1D susceptibility [29], . The locus 12q13 encompasses two genes, *ERBB3* coding for a receptor tyrosine kinase and *RPS26* coding for a ribosomal protein. *Cis* regulation of *RPS26* in diverse tissues, in particular the pancreas, has been used to argue that this gene was a more likely candidate than *ERBB3* for T1D association although this is a matter of controversy [7], [30], [31].

Module 102 contained two *cis* eQTLs associated to rs11171739, *RPS26* and *SUOX* (*P*<10^−300^ and 3.1×10^−18^, respectively). The *cis* regulation of *RPS26* in monocytes confirms that reported in other cell types [4], [7], [8]. Module 102 also contained several paralogs of *RPS26* (*RPS26L*, *RPS26L1* and *RPS26P10*) whose association with rs11171739 was probably due to cross-hybridization artifacts (Table S2). Two other genes were significantly associated in *trans*, *MADCAM1* on chromosome 19 and *CCDC4* (also known as *BEND4*) on chromosome 4, a gene of unknown function whose expression was also found associated to the 12q13 locus in leukocytes [8]. All gene expressions individually replicated for association in Cardiogenics with a proxy of rs11171739 (rs10876864, LD r^2^ = 0.91), except *RPS26L1* (*P* = 0.75) and *RPS26P10* for which there was no probe in Cardiogenics (Table S2). Moreover, all associations were in the same direction in the two studies and the SNPs were associated with very similar R^2^.

Among all the genes of module 102, *MADCAM1* (mucosal addressin cell adhesion molecule-1) appears as the strongest biological candidate for T1D [32]–[34]. However, caution is needed in the interpretation of the present results for several reasons. First, when performing the analysis at a probe level, the effect of rs11171739 was observed for only one of the two *Illumina* probes (ILMN_1767973). Second, this probe was detected (i.e. expressed above background) only in a small fraction of subjects (∼7%), the other probe being undetected. The SNP association for this probe, however, strongly replicated in Cardiogenics (Table S2), albeit that both probes were considered as “undetected” according to the detection criteria set for their analysis in Cardiogenics. Nevertheless, the consistency of the association with the SNP in both studies raises an important issue related to the difference between lack of detection and lack of expression, as recently indicated by a study showing that a large fraction of X-linked genes considered as non-expressed by microarray studies were actually detectable by RNA-sequencing quantification [35]. Even when its expression level is below the microarray detection threshold, a transcript may be of great interest if it proves to be related to a SNP or any other relevant factor. The increasing power of most contemporary transcriptomic studies should facilitate the detection of effects that were missed in earlier less-powered studies. Further validation experiments in monocytes using RT-PCR indicated hybridization problems around the *MADCAM1* exon 4 region where the associated *Illumina* probe is located and did not replicate the association (unpublished results). Since SNPs are present within the sequence used for replication (www.ensembl.org), we cannot exclude the possibility that insufficient hybridization and/or cross-hybridization affects the present results. Nevertheless, using probes and primers located in other exonic regions of the gene, high expression of *MADCAM1* in monocytes was detected. Further, unpublished expression data on exon level in peripheral blood mononuclear cells confirmed these observations of *MADCAM1* expression and association with rs11171739 (*P*<0.03).

### Association of pattern 62 with locus 12q24 involved in pleiotropic phenotypes

**Figure 3:**
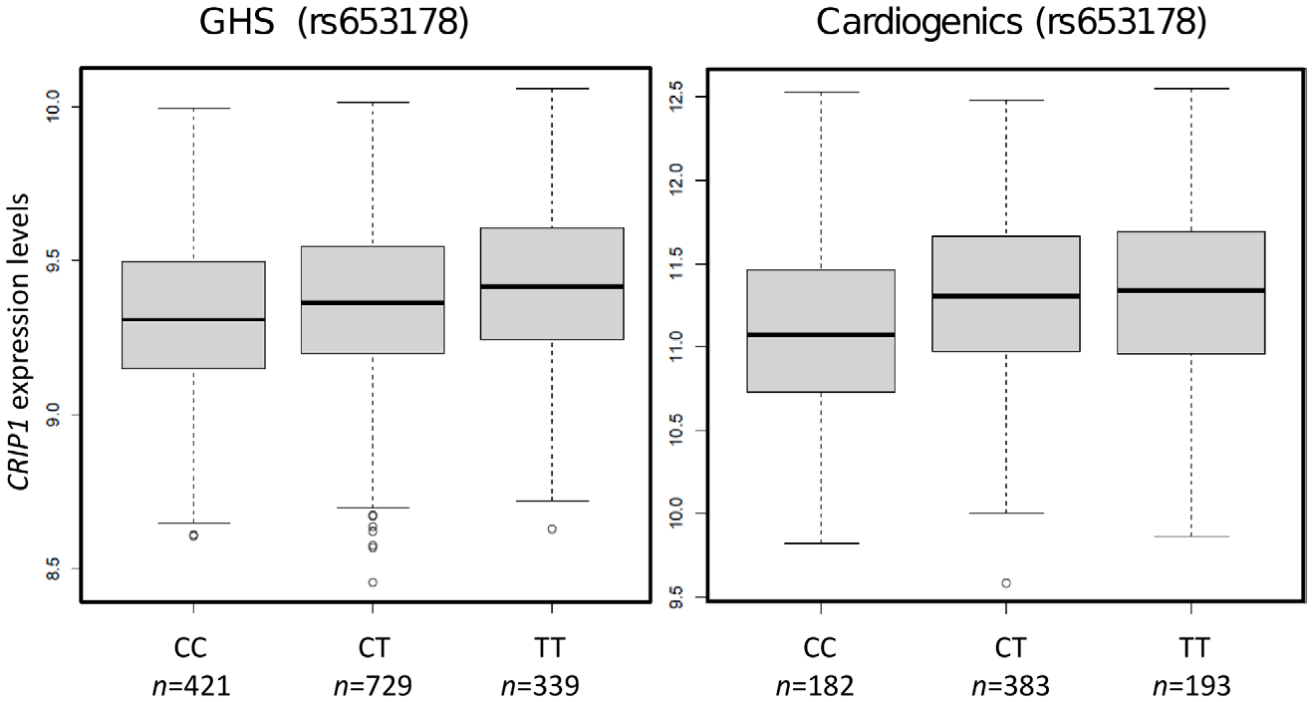
Box plots showing the association of rs653178 at locus 12q24 (*SH2B3*) with *CRIP1* expression in GHS and Cardiogenics.

The association between pattern 62 and rs653178 at locus 12q24 (*P* = 2.4×10^−9^ for association, *P* = 5.5×10^−10^ for enrichment) deserved attention for several reasons. First, the locus 12q24 has been reported in GWAS to be involved in pleiotropic phenotypes including celiac disease [36], T1D [30], asthma [37], myocardial infarction and coronary artery disease [26], [37], blood pressure (BP) [38]–[40], platelets counts [26], eosinophil number [37] and hematocrit [39]. The locus encompasses two genes, *SH2B3* and *ATXN2*, *SH2B3* being generally considered as the most likely candidate for disease susceptibility. Second, pattern 62 strongly correlated with systolic (*P* = 2.7×10^−20^) and diastolic (*P* = 5.7×10^−15^) BP in GHS subjects. Third, the most significant gene expression within module 62 was *CRIP1* (*P* = 2.8×10^−7^) which, in a previous analysis of GHS data, emerged as the strongest correlate of systolic BP [20]. The association of rs653178 with *CRIP1* expression replicated in Cardiogenics (*P* = 2.2×10^−5^). The association was in the same direction in the two studies and the SNP was associated with comparable R^2^ (2.0% in GHS and 2.6% in Cardiogenics) (Table S3, Figure 3).

*SH2B3*, also known as *LNK*, is a member of the family of adaptor proteins mediating the interaction between the extracellular receptors and intracellular signaling pathways. It is expressed in hematopoietic precursor cells and endothelial cells and acts as a broad inhibitor of growth factor and cytokine signaling pathways [41]. The association of rs653178 with pattern 62 was not mediated by a *cis* effect on *SH2B3* or by any other *cis* eQTL. In addition to *CRIP1*, module 62 included four expression traits significantly associated in *trans* with rs653178 (*RAB11FIP1*, *MYADM*, *TIPARP* and *TREM1*), among which *RAB11FIP1* showed a borderline association in Cardiogenics (*P* = 0.01) (Table S3).

Rs653178 belongs to a long-range haplotype which also carries rs3184504, a non-synonymous polymorphism (*R262W*) of the *SH2B3* gene which is located in a pleckstrin homology domain involved in intracellular signaling. This haplotype has probably arisen from a selective sweep specific to Europeans since it is not observed in African and Asian populations [26]. The *C* allele of rs653178, which is the allele associated with increased BP and higher risk of disease in GWAS, was associated with decreased expression of *CRIP1* (Figure 3). However, in the GHS population, *CRIP1* expression was positively related to SBP (*r* = 0.28) and DBP (*r* = 0.18), suggesting a complex relationship between genetic variation, gene expression and disease.

CRIP1 (cysteine-rich intestinal protein) belongs to a family of proteins with a LIM domain. LIM domains are protein interaction domains functioning in the regulation of gene expression, cell adhesion and signal transduction [42]. *CRIP1* is highly expressed in immune cells and overexpression of *CRIP1* in transgenic mice has been shown to alter the immune response [43]. *CRIP1* has also been identified as a marker of cellular proliferation in several types of cancer [44]. Consistent with this role, module 62 included several genes involved in cellular growth and/or tumorigenicity (*MYADM*, *SGMS2*, *EMP1*, *ITGA5*, *KLF6*, *FOXO1*). The present results suggest that *CRIP1* might play a central role in the pleiotropic effects of *SH2B3* in several diseases.

### Association of pattern 98 with locus 12q15 involving a large number of *trans* effects mediated by two *cis* eQTLs, *LYZ* and *YEATS4*

The strongest association was between rs11177644 at locus 12q15 and pattern 98 (*P* = 1.1×10^−92^ for association, *P* = 1.2×10^−86^ for enrichment) (Table 1). The block of association included 40 SNPs and the lead SNP explained 24.8% of the pattern variance. The module included two *cis* eQTLs, *LYZ* and *YEATS4* (48.6% and 37.7% of expression variability explained by the lead SNP, respectively) as well as 34 genes associated in *trans*, 17 of which with a *P*-value<10^−12^. Almost all associations were confirmed in Cardiogenics (Table S4). Most expression traits of module 98 negatively correlated to *LYZ* and *YEATS4* (Figure S6). When including expression levels of *LYZ* and *YEATS4* as covariates in the linear regression model relating each *trans* eQTL to rs11177644, all *trans* associations considerably decreased (median R^2^ decreasing from 3.2% to 0.5%), suggesting that these *trans* associations were mediated by *cis* regulation at the locus. *LYZ* encodes human lyzozyme which is secreted by monocytes and has a bacteriolytic function. *YEATS4* (also known as *GAS41*) is a member of a large family of domain proteins which form complexes involved in chromatin modification and transcriptional regulation and has a strong link to cancer [45]. It was not possible from the present data to infer whether pattern 98 reflects a unique pathway involving *LYZ* and *YEATS4* or whether it was a mixture of two independent pathways that showed coincidental correlation because of the physical proximity of *LYZ* and *YEATS4* on chromosome 12.

### Comparison of ICA and weighted gene co-expression network analysis (WGCNA)

To validate the approach used in this study, we compared the results obtained by ICA to those obtained by WGCNA, a method recently proposed to identify sets of co-expressed genes [46]–[48]. The WGCNA method is based on a clustering of genes into non overlapping classes, called “modules”, based on their profiles of co-expression. Each resulting module is then characterized by its first principal component referred to as the module eigengene (ME).

**Figure 4:**
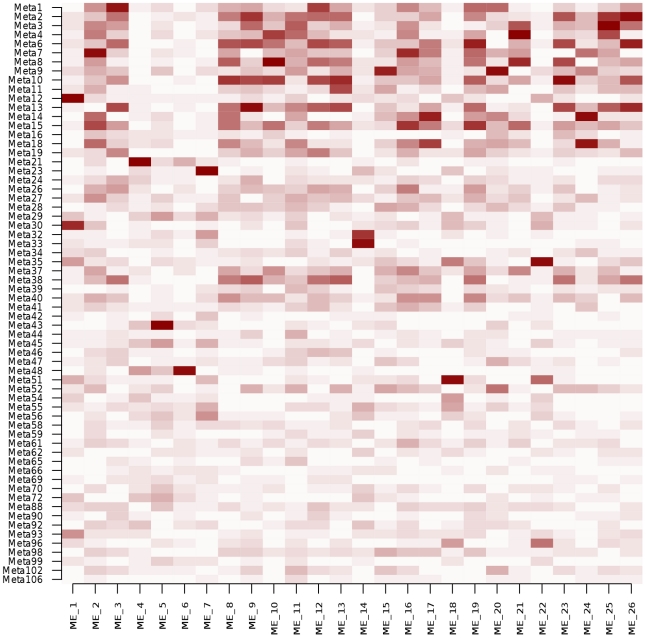
Heatmap of absolute Pearson correlation coefficients between expression patterns obtained by ICA and module eigengenes (ME) obtained by WGCNA. ICA patterns (rows) are ordered by decreasing explained variance.

When applying the WGCNA method to our data with default parameters, the 12,808 gene expression traits were clustered into 26 modules (Table S5). We computed the correlations between the 26 MEs and the 64 patterns obtained by ICA (Figure 4). Twenty-three MEs (88%) exhibited a correlation >0.8 with at least one ICA pattern. Conversely, only 20 ICA patterns (31%) correlated to a ME with the same intensity, suggesting that ICA was able to identify patterns that were not represented by WGCNA modules, such as patterns 62 and 102 described above. Eleven of the 26 original WGCNA MEs (42%) were found enriched in GO categories against 42 modules (66%) for ICA using the same significance threshold.

To get a more balanced comparison between the two methods, we increased the number of clusters extracted by WGCNA by tuning the parameters (deepSplit and minModuleSize), leading to 71 WGCNA modules (Table S6). Although the advantage of ICA appeared weaker in that case, the tendency remained the same: the fraction of WGCNA MEs exhibiting a correlation >0.8 with at least one ICA pattern was 52% (n = 37), while 42% of ICA patterns (n = 27) correlated to one of the 71 MEs (Figure S7). Only 14 (20%) of these 71 MEs were found enriched in GO categories. The higher interpretability of ICA modules in known biological functions was however mostly attributable to the larger size of ICA modules (median size: 178.5 genes) compared to WGCNA modules (median size: 46 genes). Indeed, when selecting the subset of the 200 genes the most correlated to each ME, or with the highest absolute contribution to the ICA pattern signature, respectively, the proportions of GO enriched subsets was similar between ICA (67%, n = 43) and WGCNA (63%, n = 45).

**Figure 5:**
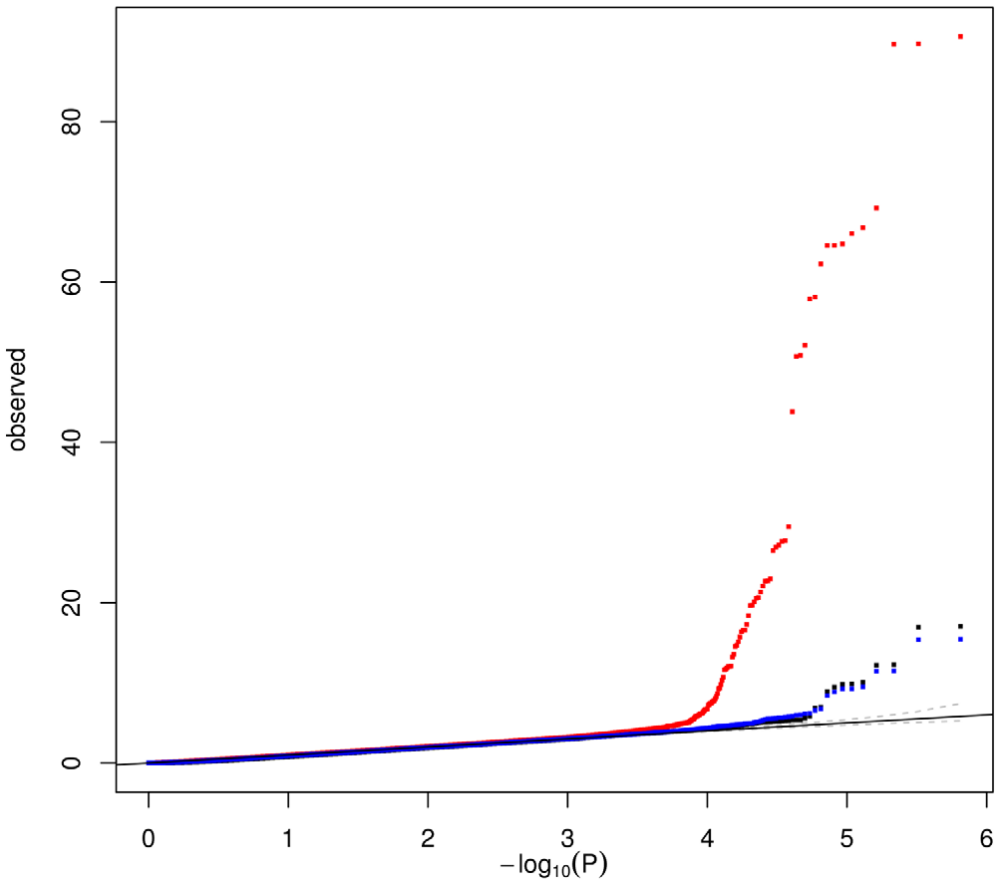
Quantile–quantile plot comparing the associations of 675,350 SNPs with patterns obtained by ICA (64 patterns – red) and module eigengenes (MEs) obtained by WGCNA with default (26 MEs – black ) or tuned parameters (71 MEs – blue). For each SNP, the best *P*-value over the 26 or 71 MEs and the 64 ICA patterns, respectively, is shown. A Sidak correction was applied to correct for the number of MEs (patterns, resp.) tested.

We next compared the power of the two methods for identifying SNPs associated with sets of co-expressed genes. Figure 5 compares the quantile-quantile plots of the Sidak-corrected *P*-values obtained when testing the 675,350 SNPs against the 26 (or the 71) WGCNA MEs on one hand, and the 64 ICA patterns on the other hand. Much stronger associations were found with ICA patterns than with WGCNA MEs, regardless of the number of modules extracted by WGCNA. Worthy of note, the strongest associations (*P*<10^−16^) detected with the 26 original WGCNA MEs involved SNPs of the *ARHGEF3* locus, which turned out to be likely explained by contamination by platelet RNA (see above).

## Discussion

Various methods have been proposed to detect sets of co-expressed genes, including nonnegative matrix factorization [49], connectivity-based approaches such as WGCNA [6], [46]–[48], [50] or Bayesian networks [7], [10], [12], [51]. The ICA method [18], [19] used in the present study is based on the assumption that the co-expression of genes may be described by a small number of latent features exerting independent influences on expression. Ideally, these features may be related to distinct biological causes of variation, like regulators of gene expression, cellular functions or response to environment [19]. ICA has been applied to different types of microarray data, in particular to identify expression signatures in cancer [52]–[54].

Most of the components extracted by ICA could be characterized by a specific module of genes. A GO enrichment analysis indicated that two thirds of these modules were enriched in GO categories, thus highlighting the ability of ICA to recover biologically meaningful covariation. The proportion of enriched modules might be artificially inflated by the fact that ICA allows for modules of overlapping gene sets, leading to similarity between modules. However, the pairwise overlap between modules was generally limited (ranging from 1 to 20%, median 6%). In addition, most of the GO categories that were shared between similar modules were relatively large categories (e.g. immune response or intracellular components) and it may be hypothesized that the corresponding modules reflected specific aspects of a more general process. As pointed out by others [13], modules can help in functional annotation of genes of unknown function based on known annotations of other genes in the module, such as *CCDC4* in the *RPS26*-associated module.

Patterns of co-expression might be confounded by systematic variations introduced during sample processing or microarray measurements and by heterogeneity of the cell population [9], [24]. In particular, patterns observed in unseparated peripheral blood mononuclear cells or whole tissues are more likely to reflect variations in the tissue composition rather than true cell-specific co-expression. In the present study, monocytes were isolated by negative selection. The choice of the method for separation of leukocytes is a matter of debate. Negative selection results in lower cell purity, while positive selection may induce cellular activation and altered transcription due to cross-linking cell surface antigens. A comparison of the two methods in 6 subjects suggested that positive selection did not induce important changes in gene expression [24]. However, the study had little power to detect modest variations such as those involved in *trans* associations. Thanks to the recent advances in the characterization of genes specific of the different blood cell lineages [25], it is now possible to better control *in silico* for potential heterogeneity of the cell population under study. We used this information to test the robustness of the SNP-pattern associations after adjustment for surrogate variables of contamination (S. Maouche *et al. In preparation*). It was not possible, however, to adjust expression data prior to ICA since we observed that such adjustment could induce other artifactual correlations, probably because genes supposed to be specific of a given cell type may also be expressed, although at lower levels, in the monocyte. Since none of the presently available methods yields an 100% purity, *in silico* adjustment appears as a solution for *post hoc* controlling the robustness of associations as recently proposed [55], [56].

Using this robust approach, we investigated *trans* associations of pattern of co-expression in a large population-based study, the Gutenberg Health Study (GHS). We replicated significant *tran*s associations (but not entire modules) detected in the GHS in an independent study, Cardiogenics. Our results showed three genomic regions associated in *trans* with modules of co-expressed genes. For two of these regions, the *trans* effects appeared to be mediated by one (or two) *cis* eQTLs (*RPS26* and *LYZ/YEATS4*) while in the third case (*SH2B3*), the *trans* associations were likely to be explained by an alteration of the intracellular signaling. The biological hypotheses raised by these findings will have to be replicated in further experimental studies.

In conclusion, the present study shows that a method exploiting the structure of co-expressions among genes such as ICA can help identify genomic regions involved in *trans* regulation of sets of genes and provide clues for understanding the mechanisms linking GWAS loci to disease. It also suggests that *trans* associations involving large sets of gene expressions may reflect stratification of the cell population that can be controlled for by *in silico* adjustment.

## Methods

More details are provided in Text S1.

### Subjects

Study participants of both sexes aged 35–74 yr, were successively enrolled into the GHS, a community-based, prospective, observational single-center cohort study in the Rhein-Main region in western mid-Germany. The majority of participants were of European origin. A few non-European individuals detected by MDS analysis of genetic data (see below) were excluded prior to analysis, leaving 1,490 subjects for further analysis.

### Ethics statement

All subjects gave written informed consent. Ethical approval was given by the local ethics committee and by the local and federal data safety commissioners.

### Genotyping

GWV genotyping was performed using the *Affymetrix* Genome-Wide Human SNP Array 6.0 and the Genome-Wide Human SNP *Nsp*I/*Sty*I 5.0 Assay kit. Genotypes were called using the *Affymetrix* Birdseed-V2 calling algorithm and quality control was performed using GenABEL [57] (http://mga.bionet.nsc.ru/nlru/GenABEL/).

### Separation of monocytes

Separation of monocytes was conducted within 60 min after blood collection. 8 mL blood was collected using the Vacutainer CPT Cell Preparation Tube System (BD, Heidelberg, Germany) and 400 µL RosetteSep Monocyte Enrichment Cocktail (StemCell Technologies, Vancouver, Canada) was added immediately after blood collection. This cocktail contains antibodies directed against cell surface antigens on human hematopoietic cells (CD2, CD3, CD8, CD19, CD56, CD66b) and glycophorin A on red blood cells. Total RNA was extracted the same day using Trizol extraction and purification by silica-based columns.

### Microarray hybridization and data pre-processing

GWE assessment was performed using the *Illumina* HT-12 v3 BeadChip. Pre-processing of data and quantile normalization was performed using *Beadstudio*. Analysis was performed on the mean levels of probes of genes. To stabilize variance across gene expression levels, data were arcsinh-transformed. The *Illumina* HT-12 chip included 37,804 genes (including probes not assigned to RefSeq transcripts). A gene was declared expressed when the fraction of samples with a detection *P*-value<0.05 for that gene was significantly higher than 5% (Text S1). After removing putative and/or non well characterized genes (i.e. gene names starting by KIAA, FLJ, HS., C*x*orf, MGC, LOC, NT_, ENSG), 12,808 genes remained for analysis.

### Outliers

Multi-dimensional scaling (MDS) was performed on GWE and GWV datasets and outliers in either dataset were excluded from analyses (Text S1, Figures S1, S2, S3, S4).

### Independent component analysis (ICA)

After normalization, the distribution of each expression trait across individuals was centred and standardized. The R function *svd* was used prior to ICA to reduce the dimensionality of data and determine the optimal number of patterns to be extracted by ICA (Text S1). ICA was performed with the R *fastICA* algorithm which uses negentropy to minimize the dependency between components. The algorithm was configured using parallel extraction method and *logcosh* approximation of negentropy with α = 1. To avoid trapping in a local maximum, 10 runs of the algorithm were performed and the run with the maximal negentropy was kept.

### Definition of modules and enrichment analyses

The *fdrtool* R package [23] was used to define the subset of genes characterizing each signature (“module”). The statistics to which the method was applied was the entry *s_ik_* of matrix *S*, considered as a normal score. The signature of each component was modeled as a mixture of two distributions (null and alternative). The method fits a null (Gaussian) distribution around the median of the signature distribution. A gene *i* was considered as belonging to the module of the signature *k* if *s_ik_* had a probability <10^−3^ of being drawn under the null (FDR<10^−3^).

Functional annotations were made using the Gene Ontology database. Module enrichment was tested using a hypergeometric test. A threshold of 5.45×10^−6^ correcting for the number of categories tested was taken to declare that a category was significantly enriched in genes from a module.

Pairwise overlap between modules was defined, for two modules A and B, as the ratio between the number of genes shared by A and B to the total number of genes belonging to A or B.

### Testing association of patterns with genotype

Association of the 64 patterns with the 675,350 SNPs was first tested by ANOVA with 2 d.f. using the C variance program of the GNU library TAMU_ANOVA (www.stat.tamu.edu/~aredd/tamuanova/). In this first step, a *P*-value<10^−7^ was considered as suggestive. For suggestive SNP-pattern associations, we tested in a second step the enrichment of the module in expressions individually associated to the SNP by ANOVA at *P*<10^−5^. For this second test, we used a hypergeometric test with a study-wise threshold of significance of 1.15×10^−9^ (Bonferroni-corrected for 64 modules×675,350 SNPs).

### Adjustment for potential contamination by non-monocytic cells

For each blood cell type (CD4+, CD8+, CD19+, CD56+, CD66b+, erythroblasts and megakaryocytes), we listed from the HaemAtlas [25] the genes reported as specific of that lineage (Table S5 from [25]). Expression levels of the cell-specific genes were averaged in each subject and taken as a surrogate variable of the degree of contamination by each cell type (S. Maouche *et al. in preparation*). Genes were considered specific from one lineage when they were over-expressed with a fold change higher than 2 in the considered lineage compared to all others [25]. In every GHS sample, the degree of contamination by a given cell type was assessed by averaging the expression levels of the subset of the cell-specific genes in that sample. This resulted in 7 surrogate variables for contamination. All significant SNP-pattern associations were re-tested by simultaneously including these 7 variables as covariates in the regression linear model.

### Replication in Cardiogenics

The population study included 363 patients with coronary artery disease recruited in Lübeck and Regensburg (Germany), Leicester (UK) and Paris (France) and 395 healthy individuals recruited in Cambridge (UK) within the Cardiogenics Consortium (http://www.cardiogenics.eu). All subjects were of European descent (Text S1). Genome-wide genotyping was carried out using the *Illumina* Sentrix Human Custom 1.2 M array and the Human 610 Quad Custom array. Monocytes were isolated from whole blood using CD14 micro beads (*Miltenyi*). Gene expression profiling was performed using Human Ref-8 Sentrix Bead Chip arrays (*Illumina*). Pre-processing of data and statistical analysis were performed in the R statistical environment. For the genes to be replicated, we did not apply any filtering on the level of detection since the detection power was lower in Cardiogenics than in GHS and some genes might be missed for that reason. Association of gene expression with genotype was tested by analysis of variance with adjustment on age, gender and center. Analysis was performed at the probe level and the probes showing the strongest association were selected. The association with a module was considered as replicated when the two following criteria were met: 1) at least two genes were significantly associated to the SNP at a threshold of 0.05 after Bonferroni correction for the number of genes present in the module; 2) the number of genes associated to the SNP at a 0.05 threshold was significantly higher than 5%, based on a binomial distribution. This implies that association with the module was considered replicated even when not all gene-specific associations were replicated. Full association results from the replication are available at the GHS_ICA_Modules database. For each replicated module, associations in Cardiogenics are reported in Tables S2, S3 and S4 for all expression traits associated at *P*<10^−6^ in GHS. For each gene, the probe showing the strongest association is reported.

### Weighted gene co-expression network analysis (WGCNA)

WGCNA was performed on normalized expression data using the *blockwiseModules* function from the WGCNA R package (v0.92). The TOM matrix was computed from the whole set of 12,808 gene expressions (maxblocksize was set to 12,808) and all other tuning parameters were set to their default value (including dynamic tree cutting and automated merging of close modules). Module eigengenes (MEs) were computed by the *blockwiseModules* function as the first principal component of each module. In order to increase the number of clusters to get a more balanced comparison with ICA, a second run of the WGCNA algorithm was performed with parameters deepSplit = 4 and minModuleSize = 10 (size of the smallest ICA module: n = 14).

### Comparison of ICA and WGCNA methods

Pairwise Pearson correlation coefficients were computed between the 64 patterns obtained by ICA and the 26 (or 71) MEs obtained by WGCNA. To compare the power of the two methods to detect associations with SNPs, we tested the association of the 675,350 SNPs with the 26 (or 71) WGCNA MEs by linear regression analysis assuming an additive allele effect. For each SNP, we retained the best *P*-value over the 26 (or 71) MEs and applied a Sidak correction for the number of WGCNA MEs tested. The same analysis was performed with the 64 ICA patterns. QQ plots of the 675,350 corrected *P*-values were displayed for the three methods (ICA, WGCNA with defaults parameters, WGCNA with tuned parameters).

### GHS_Express

A downloadable SQL database compiling the results of the various associations between SNPs and expression traits is available online (http://genecanvas.ecgene.net/uploads/ForReview/). For using this database, see Methods S1 in [20].

### GHS_ICA_modules

More detailed results of the analyses performed in the present study are compiled in an HTML database that is available online (http://genecanvas.ecgene.net/uploads/ForReview/). These results include correlations between patterns, module composition and enrichment, associations between SNPs and individual expression traits within modules in GHS and Cardiogenics.

## Supporting Information

Figure S1 Checking for outliers or population stratification from GWV data in GHS – Run 1. The figure plots the coordonates of all subjects on the first 2 principal components otained by MDS analysis of a matrix of pairwise IBS values between subjects. After this first run, 17 outliers (red circles) were excluded.
(TIF)

Figure S2 Checking for outliers or population stratification from GWV data in GHS – Run 2. A second run of the MDS analysis was performed after exclusion of the 17 outliers identified in run 1. After this second run, 54 additional subjects (red circles) were excluded.
(TIF)

Figure S3 Checking for outliers or population stratification from GWV data in GHS – Run 3. The third run of MDS analysis shows that the remaining population is genetically homogeneous.
(TIF)

Figure S4 Checking for outliers from GWE data in GHS. MDS analysis was applied on a matrix of pairwise distances between subjects calculated as 1 minus the absolute correlation between arrays. Ten subjects (red circles) were excluded from analysis.
(TIF)

Figure S5 Screeplot from the singular value decomposition (SVD) analysis of the matrix of 12,808 expressions×1,490 subjects in GHS. The screeplot plots the variances explained by the principal components of the SVD. The blue solid curve shows the individual variance explained by the *s* ^th^ component (*s* ^th^ eigenvalue) and the green curve shows the cumulative variance explained by the first *s* components on the real data matrix. The brown dashed line corresponds to the eigenvalues obtained from a SVD on a random matrix obtained by permuting the 1,490 subjects independently for each gene expression. The purple solid line was obtained from the same random matrix but the *s* ^th^ eigenvalue was corrected for the variance explained by the (*s*-1) first components by subtracting, for each of the preceding component, the excess variance explained by the component (difference between real and random eigen values) from the remaining eigenvalues. The optimal number of components was determined at the intersection between the blue curve (observed variance) and the purple curve (variance expected under random after having already extracted (*s*-1) components), considering that beyond this number, components mostly reflected noise. The optimal number was 112 (red vertical line).
(TIF)

Figure S6 Heatmap of pairwise correlations among the 34 gene expressions of module 98. Correlations are shown before and after adjustment for the associated SNP rs11177644. The two *cis* eQTLs, *LYZ* and *YEATS4*, are shown in first. Positive correlations are shown in red, negative ones in blue.
(TIF)

Figure S7 Heatmap of absolute Pearson correlation coefficients between expression patterns obtained by ICA and the 71 module eigengenes (ME) obtained by WGCNA with tuned parameters: deepSplit = 4, minModuleSize = 10. ICA patterns (rows) are ordered by decreasing explained variance.
(PDF)

Table S1 List of the modules characterizing the 64 signatures obtained by ICA and enrichment of these modules in GO categories.
(DOC)

Table S2 Module102-rs11171739(rs10876864).
(TXT)

Table S3 Module62-rs653178(rs653178).
(TXT)

Table S4 Module98-rs11177644(rs6581889).
(TXT)

Table S5 List of the 26 modules obtained by WGCNA with default parameters and enrichment of these modules in GO categories.
(DOC)

Table S6 List of the 71 modules obtained by WGCNA with tuned parameters and enrichment of these modules in GO categories.
(DOC)

Text S1 Supporting information: further details of Methods, and members of the Cardiogenics Consortium.
(DOC)

## References

[1] Manolio TA, Collins FS, Cox NJ, Goldstein DB, Hindorff LA (2009). Finding the missing heritability of complex diseases.. Nature.

[2] Cookson W, Liang L, Abecasis G, Moffatt M, Lathrop M (2009). Mapping complex disease traits with global gene expression.. Nat Rev Genet.

[3] Goring HH, Curran JE, Johnson MP, Dyer TD, Charlesworth J (2007). Discovery of expression QTLs using large-scale transcriptional profiling in human lymphocytes.. Nat Genet.

[4] Dixon AL, Liang L, Moffatt MF, Chen W, Heath S (2007). A genome-wide association study of global gene expression.. Nat Genet.

[5] Stranger BE, Nica AC, Forrest MS, Dimas A, Bird CP (2007). Population genomics of human gene expression.. Nat Genet.

[6] Emilsson V, Thorleifsson G, Zhang B, Leonardson AS, Zink F (2008). Genetics of gene expression and its effect on disease.. Nature.

[7] Schadt EE, Molony C, Chudin E, Hao K, Yang X (2008). Mapping the genetic architecture of gene expression in human liver.. PLoS Biol.

[8] Idaghdour Y, Czika W, Shianna KV, Lee SH, Visscher PM (2010). Geographical genomics of human leukocyte gene expression variation in southern Morocco.. Nat Genet.

[9] Kang HM, Ye C, Eskin E (2008). Accurate discovery of expression quantitative trait loci under confounding from spurious and genuine regulatory hotspots.. Genetics.

[10] Schadt EE, Zhang B, Zhu J (2009). Advances in systems biology are enhancing our understanding of disease and moving us closer to novel disease treatments.. Genetica.

[11] Yvert G, Brem RB, Whittle J, Akey JM, Foss E (2003). Trans-acting regulatory variation in Saccharomyces cerevisiae and the role of transcription factors.. Nat Genet.

[12] Zhu J, Zhang B, Smith EN, Drees B, Brem RB (2008). Integrating large-scale functional genomic data to dissect the complexity of yeast regulatory networks.. Nat Genet.

[13] Ayroles JF, Carbone MA, Stone EA, Jordan KW, Lyman RF (2009). Systems genetics of complex traits in Drosophila melanogaster.. Nat Genet.

[14] Mehrabian M, Allayee H, Stockton J, Lum PY, Drake TA (2005). Integrating genotypic and expression data in a segregating mouse population to identify 5-lipoxygenase as a susceptibility gene for obesity and bone traits.. Nat Genet.

[15] Schadt EE, Lamb J, Yang X, Zhu J, Edwards S (2005). An integrative genomics approach to infer causal associations between gene expression and disease.. Nat Genet.

[16] Ghazalpour A, Doss S, Zhang B, Wang S, Plaisier C (2006). Integrating genetic and network analysis to characterize genes related to mouse weight.. PLoS Genet.

[17] Biswas S, Storey JD, Akey JM (2008). Mapping gene expression quantitative trait loci by singular value decomposition and independent component analysis.. BMC Bioinformatics.

[18] Hyvarinen A, Oja E (2000). Independent component analysis: algorithms and applications.. Neural Netw.

[19] Liebermeister W (2002). Linear modes of gene expression determined by independent component analysis.. Bioinformatics.

[20] Zeller T, Wild P, Szymczak S, Rotival M, Schillert A (2010). Genetics and beyond—The transcriptome of human monocytes and disease susceptibility.. PLoS ONE.

[21] Lee SI, Batzoglou S (2003). Application of independent component analysis to microarrays.. Genome Biol.

[22] Carpentier AS, Riva A, Tisseur P, Didier G, Henaut A (2004). The operons, a criterion to compare the reliability of transcriptome analysis tools: ICA is more reliable than ANOVA, PLS and PCA.. Comput Biol Chem.

[23] Strimmer K (2008). A unified approach to false discovery rate estimation.. BMC Bioinformatics.

[24] Lyons PA, Koukoulaki M, Hatton A, Doggett K, Woffendin HB (2007). Microarray analysis of human leucocyte subsets: the advantages of positive selection and rapid purification.. BMC Genomics.

[25] Watkins NA, Gusnanto A, de Bono B, De S, Miranda-Saavedra D (2009). A HaemAtlas: characterizing gene expression in differentiated human blood cells.. Blood.

[26] Soranzo N, Spector TD, Mangino M, Kuhnel B, Rendon A (2009). A genome-wide meta-analysis identifies 22 loci associated with eight hematological parameters in the HaemGen consortium.. Nat Genet.

[27] Sato S, Sanjo H, Takeda K, Ninomiya-Tsuji J, Yamamoto M (2005). Essential function for the kinase TAK1 in innate and adaptive immune responses.. Nat Immunol.

[28] Heinig M, Petretto E, Wallace C, Bottolo L, Rotival M (2010). A conserved trans-acting regulatory locus underlies a proinflammatory gene expression network and susceptibility to autoimmune type 1 diabetes.. Nature.

[29] The Wellcome Trust Case Control Consortium (2007). Genome-wide association study of 14,000 cases of seven common diseases and 3,000 shared controls.. Nature.

[30] Todd JA, Walker NM, Cooper JD, Smyth DJ, Downes K (2007). Robust associations of four new chromosome regions from genome-wide analyses of type 1 diabetes.. Nat Genet.

[31] Plagnol V, Smyth DJ, Todd JA, Clayton DG (2009). Statistical independence of the colocalized association signals for type 1 diabetes and RPS26 gene expression on chromosome 12q13.. Biostatistics.

[32] Hanninen A, Taylor C, Streeter PR, Stark LS, Sarte JM (1993). Vascular addressins are induced on islet vessels during insulitis in nonobese diabetic mice and are involved in lymphoid cell binding to islet endothelium.. J Clin Invest.

[33] Hanninen A, Jaakkola I, Jalkanen S (1998). Mucosal addressin is required for the development of diabetes in nonobese diabetic mice.. J Immunol.

[34] Phillips JM, Haskins K, Cooke A (2005). MAdCAM-1 is needed for diabetes development mediated by the T cell clone, BDC-2.5.. Immunology.

[35] Xiong Y, Chen X, Chen Z, Wang X, Shi S RNA sequencing shows no dosage compensation of the active X-chromosome.. Nat Genet.

[36] Hunt KA, Zhernakova A, Turner G, Heap GA, Franke L (2008). Newly identified genetic risk variants for celiac disease related to the immune response.. Nat Genet.

[37] Gudbjartsson DF, Bjornsdottir US, Halapi E, Helgadottir A, Sulem P (2009). Sequence variants affecting eosinophil numbers associate with asthma and myocardial infarction.. Nat Genet.

[38] Newton-Cheh C, Johnson T, Gateva V, Tobin MD, Bochud M (2009). Genome-wide association study identifies eight loci associated with blood pressure.. Nat Genet.

[39] Ganesh SK, Zakai NA, van Rooij FJ, Soranzo N, Smith AV (2009). Multiple loci influence erythrocyte phenotypes in the CHARGE Consortium.. Nat Genet.

[40] Levy D, Ehret GB, Rice K, Verwoert GC, Launer LJ (2009). Genome-wide association study of blood pressure and hypertension.. Nat Genet.

[41] Velazquez L, Cheng AM, Fleming HE, Furlonger C, Vesely S (2002). Cytokine signaling and hematopoietic homeostasis are disrupted in Lnk-deficient mice.. J Exp Med.

[42] Kadrmas JL, Beckerle MC (2004). The LIM domain: from the cytoskeleton to the nucleus.. Nat Rev Mol Cell Biol.

[43] Lanningham-Foster L, Green CL, Langkamp-Henken B, Davis BA, Nguyen KT (2002). Overexpression of CRIP in transgenic mice alters cytokine patterns and the immune response.. Am J Physiol Endocrinol Metab.

[44] Hao J, Serohijos AW, Newton G, Tassone G, Wang Z (2008). Identification and rational redesign of peptide ligands to CRIP1, a novel biomarker for cancers.. PLoS Comput Biol.

[45] Schulze JM, Wang AY, Kobor MS (2009). YEATS domain proteins: a diverse family with many links to chromatin modification and transcription.. Biochem Cell Biol.

[46] Keller MP, Choi Y, Wang P, Davis DB, Rabaglia ME (2008). A gene expression network model of type 2 diabetes links cell cycle regulation in islets with diabetes susceptibility.. Genome Res.

[47] Chen Y, Zhu J, Lum PY, Yang X, Pinto S (2008). Variations in DNA elucidate molecular networks that cause disease.. Nature.

[48] Plaisier CL, Horvath S, Huertas-Vazquez A, Cruz-Bautista I, Herrera MF (2009). A systems genetics approach implicates USF1, FADS3, and other causal candidate genes for familial combined hyperlipidemia.. PLoS Genet.

[49] Brunet JP, Tamayo P, Golub TR, Mesirov JP (2004). Metagenes and molecular pattern discovery using matrix factorization.. Proc Natl Acad Sci U S A.

[50] Grieve IC, Dickens NJ, Pravenec M, Kren V, Hubner N (2008). Genome-wide co-expression analysis in multiple tissues.. PLoS ONE.

[51] Friedman N, Linial M, Nachman I, Pe'er D (2000). Using Bayesian networks to analyze expression data.. J Comput Biol.

[52] Zhang XW, Yap YL, Wei D, Chen F, Danchin A (2005). Molecular diagnosis of human cancer type by gene expression profiles and independent component analysis.. Eur J Hum Genet.

[53] Teschendorff AE, Journee M, Absil PA, Sepulchre R, Caldas C (2007). Elucidating the altered transcriptional programs in breast cancer using independent component analysis.. PLoS Comput Biol.

[54] Lutter D, Ugocsai P, Grandl M, Orso E, Theis F (2008). Analyzing M-CSF dependent monocyte/macrophage differentiation: expression modes and meta-modes derived from an independent component analysis.. BMC Bioinformatics.

[55] Shen-Orr SS, Tibshirani R, Khatri P, Bodian DL, Staedtler F (2010). Cell type-specific gene expression differences in complex tissues.. Nat Methods.

[56] Abbas AR, Wolslegel K, Seshasayee D, Modrusan Z, Clark HF (2009). Deconvolution of blood microarray data identifies cellular activation patterns in systemic lupus erythematosus.. PLoS ONE.

[57] Aulchenko YS, Ripke S, Isaacs A, van Duijn CM (2007). GenABEL: an R library for genome-wide association analysis.. Bioinformatics.

