# Supplementary figures and images for: Integrating Genome-Wide Genetic Variations and Monocyte Expression Data Reveals Trans-Regulated Gene Modules in Humans
Source: PLoS Genet. 2011 Dec 1;7(12):e1002367. doi: 10.1371/journal.pgen.1002367 (PMC3228821; doi:10.1371/journal.pgen.1002367)

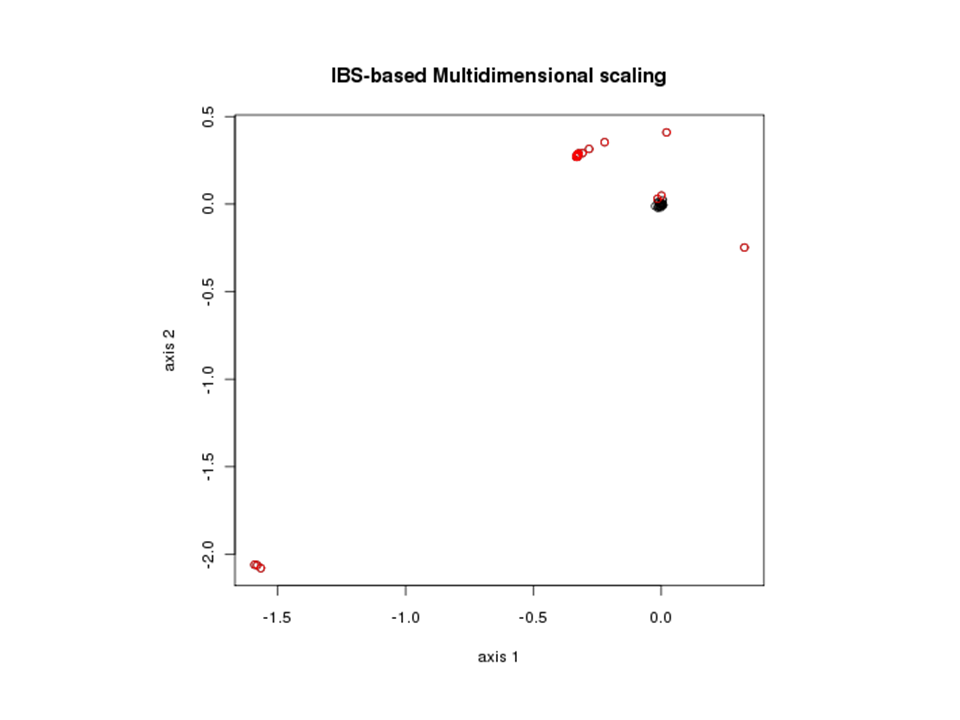

Supplement: Figure S1 — Checking for outliers or population stratification from GWV data in GHS – Run 1. The figure plots the coordonates of all subjects on the first 2 principal components otained by MDS analysis of a matrix of pairwise IBS values between subjects. After this first run, 17 outliers (red circles) were excluded. (TIF) [file pgen.1002367.s001.tif]

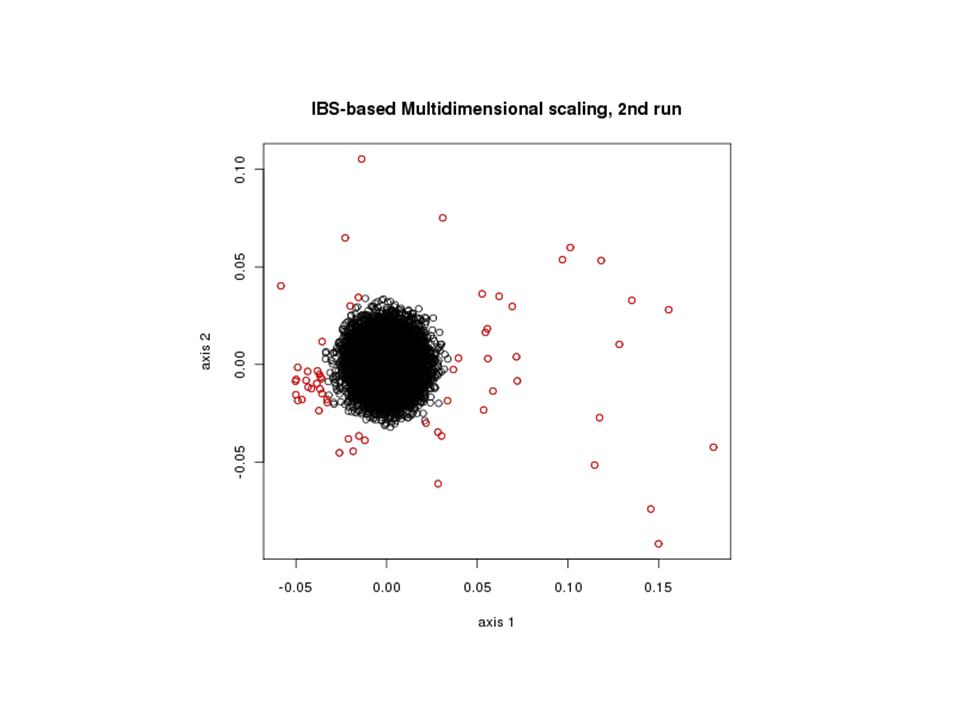

Supplement: Figure S2 — Checking for outliers or population stratification from GWV data in GHS – Run 2. A second run of the MDS analysis was performed after exclusion of the 17 outliers identified in run 1. After this second run, 54 additional subjects (red circles) were excluded. (TIF) [file pgen.1002367.s002.tif]

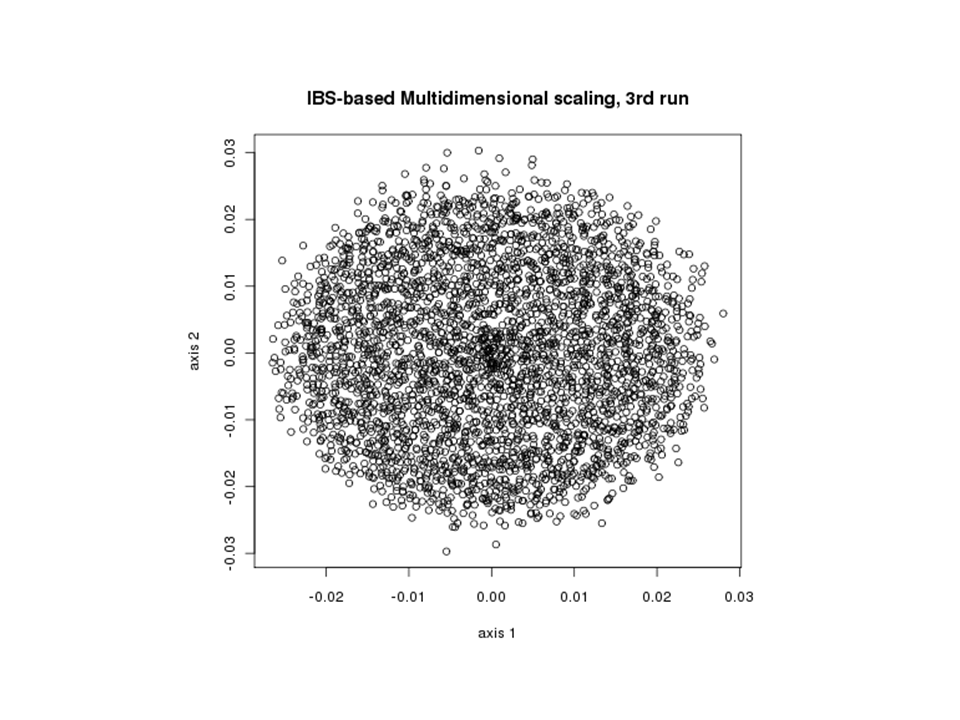

Supplement: Figure S3 — Checking for outliers or population stratification from GWV data in GHS – Run 3. The third run of MDS analysis shows that the remaining population is genetically homogeneous. (TIF) [file pgen.1002367.s003.tif]

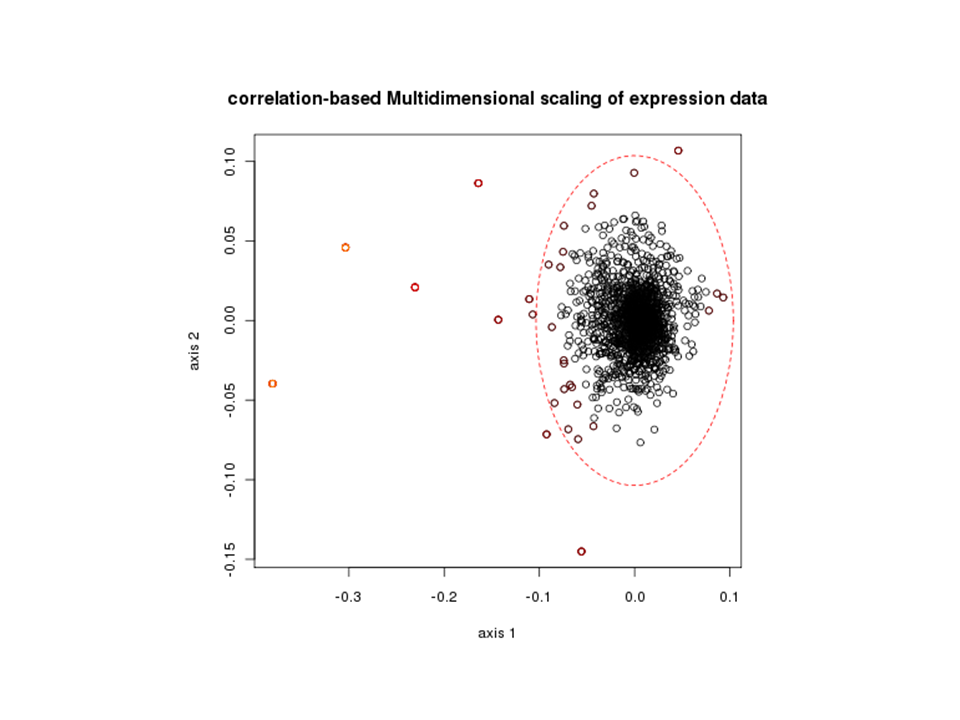

Supplement: Figure S4 — Checking for outliers from GWE data in GHS. MDS analysis was applied on a matrix of pairwise distances between subjects calculated as 1 minus the absolute correlation between arrays. Ten subjects (red circles) were excluded from analysis. (TIF) [file pgen.1002367.s004.tif]

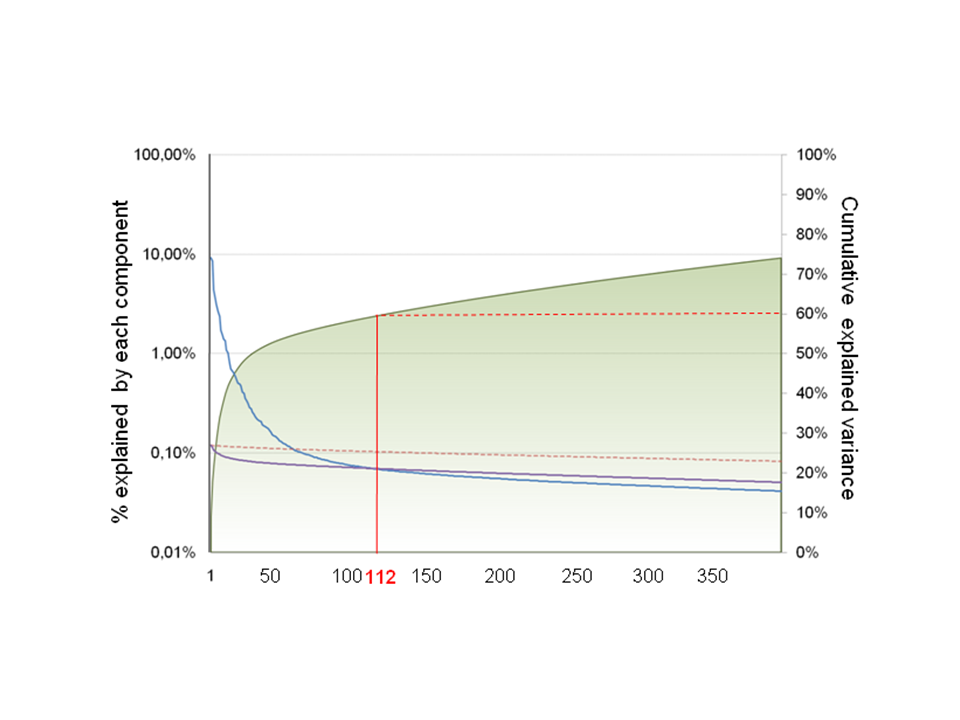

Supplement: Figure S5 — Screeplot from the singular value decomposition (SVD) analysis of the matrix of 12,808 expressions×1,490 subjects in GHS. The screeplot plots the variances explained by the principal components of the SVD. The blue solid curve shows the individual variance explained by the s th component (s th eigenvalue) and the green curve shows the cumulative variance explained by the first s components on the real data matrix. The brown dashed line corresponds to the eigenvalues obtained from a SVD on a random matrix obtained by permuting the 1,490 subjects independently for each gene expression. The purple solid line was obtained from the same random matrix but the s th eigenvalue was corrected for the variance explained by the (s-1) first components by subtracting, for each of the preceding component, the excess variance explained by the component (difference between real and random eigen values) from the remaining eigenvalues. The optimal number of components was determined at the intersection between the blue curve (observed variance) and the purple curve (variance expected under random after having already extracted (s-1) components), considering that beyond this number, components mostly reflected noise. The optimal number was 112 (red vertical line). (TIF) [file pgen.1002367.s005.tif]

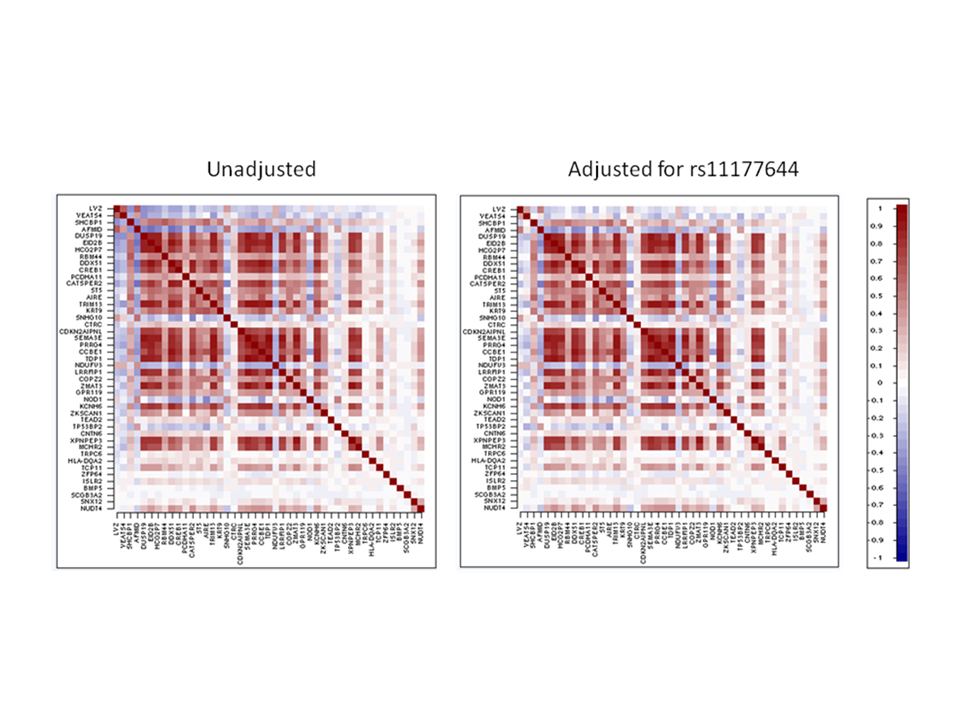

Supplement: Figure S6 — Heatmap of pairwise correlations among the 34 gene expressions of module 98. Correlations are shown before and after adjustment for the associated SNP rs11177644. The two cis eQTLs, LYZ and YEATS4, are shown in first. Positive correlations are shown in red, negative ones in blue. (TIF) [file pgen.1002367.s006.tif]

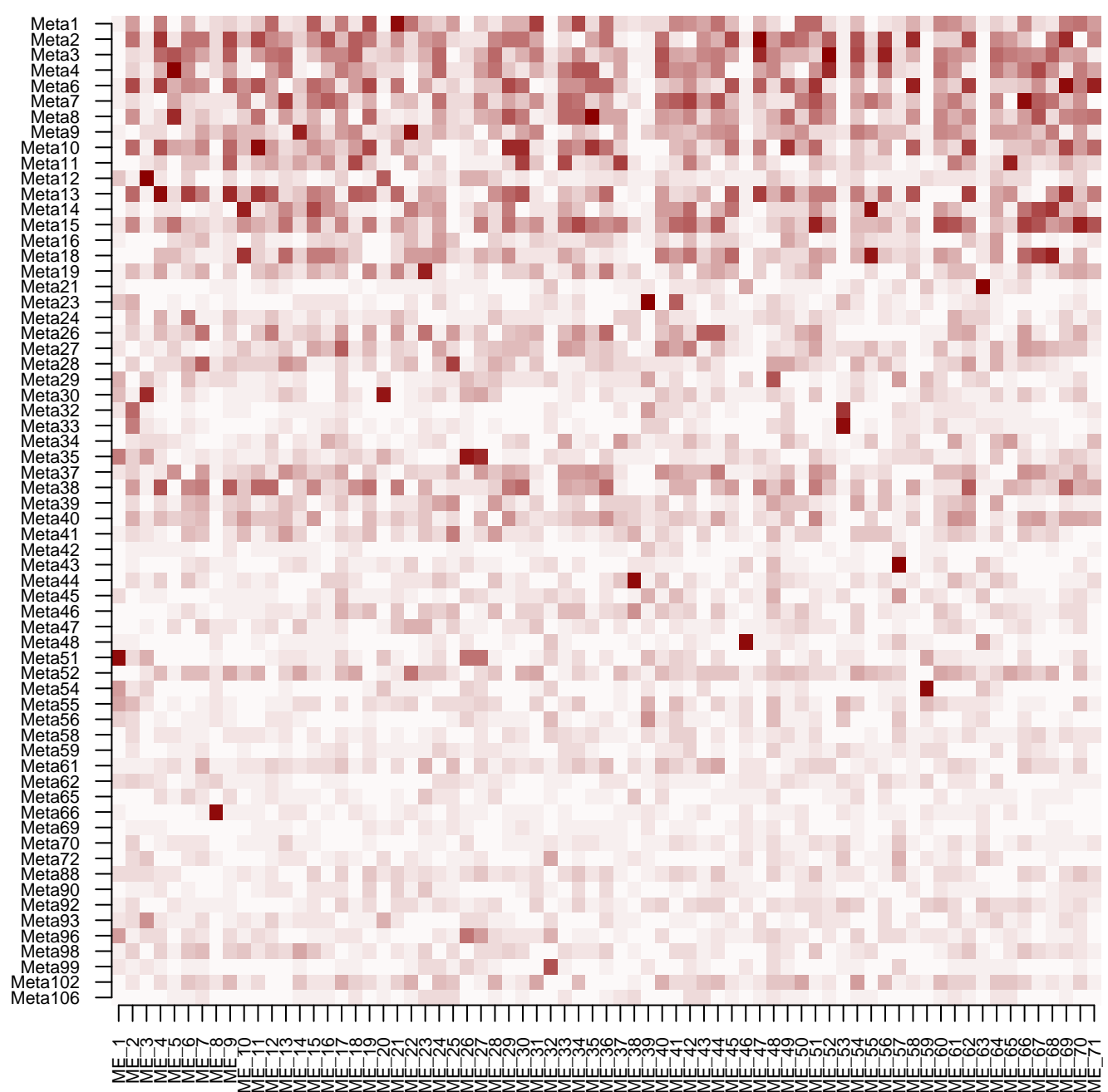

Supplement: Figure S7 — Heatmap of absolute Pearson correlation coefficients between expression patterns obtained by ICA and the 71 module eigengenes (ME) obtained by WGCNA with tuned parameters: deepSplit = 4, minModuleSize = 10. ICA patterns (rows) are ordered by decreasing explained variance. (PDF) [file pgen.1002367.s007.pdf]
