# Supplementary material for: Integrating Genome-Wide Genetic Variations and Monocyte Expression Data Reveals Trans-Regulated Gene Modules in Humans
Source: PLoS Genet. 2011 Dec 1;7(12):e1002367. doi: 10.1371/journal.pgen.1002367 (PMC3228821; doi:10.1371/journal.pgen.1002367)
Supplement: Table S1 — List of the modules characterizing the 64 signatures obtained by ICA and enrichment of these modules in GO categories. (DOC) [file pgen.1002367.s008.doc]

**Table S1. List of the modules characterizing the 64 signatures obtained by ICA and enrichment of these modules in GO categories**

| **Module** | **Number of genes in the module** | **Variance of the pattern explained by genes of the module** | **10 most significant genes in the module (most extreme in the signature distribution)** | **Significantly enriched GO categories**  **(only the 10 most significant categories are reported when**  **more than 10 have reached Bonferroni significance)** |
| --- | --- | --- | --- | --- |
| 1 | 383 | 47% | *ANXA2P1, CCAR1, PTMA, GIMAP2, LIN7C, TLR1, PPP1R15B, CLTC, SUMO1P3, PABPC3* | intracellular (p=1.9e-06) / intracellular part (p=3.1e-06) |
| 2 | 289 | 39% | *COX6B1, EEF1A1, S100A4, DAD1, RPL27, RPL17, RPS29, UBB, PITPNC1, CXCR4* | structural constituent of ribosome (p=1.7e-09) / ribonucleoprotein complex (p=3.8e-08) / ribosome (p=5.9e-08) / intracellular organelle (p=1.7e-07) / translation (p=1.2e-06) |
| 3 | 366 | 47% | *RPS6, RPL41, RPL31, STX8, ZC3H7A, PIAS1, CCT2, NDUFS5, CDV3, SAP130* | intracellular (p=1e-07) / intracellular part (p=4e-07) |
| 4 | 396 | 52% | *RPL35A, RPL26, SFRS2, PSMD10, RPS27, FUCA2, RPL21, TIAL1, H3F3B, ATP6V1B2* |  |
| 6 | 283 | 39% | *EIF4A1, TMED10, DYNLL1, SRRM1L, AMICA1, RPL12, ZNRD1, TUBA1C, PDRG1, USP10* | intracellular organelle part (p=5.2e-08) |
| 7 | 189 | 26% | *ARHGEF1, MAML1, ZFAND3, LARP5, CEBPA, LTBR, FAM53B, EIF4G1, FLII, HELZ* |  |
| 8 | 362 | 55% | *ABHD10, SLC46A3, DDX1, TBC1D15, NDUFB3, ZNF451, ZNF266, LSM3, CD46, SEC23IP* | intracellular part (p=7.6e-11) / intracellular membrane-bounded organelle (p=4.9e-10) / membrane-bounded organelle (p=5.6e-10) / intracellular (p=1.9e-09) / intracellular organelle part (p=1e-08) / intracellular organelle (p=1.4e-08) / nuclear lumen (p=7.3e-08) / nuclear part (p=1e-07) / nucleoplasm (p=2.4e-07) / nucleic acid binding (p=3.9e-07) |
| 9 | 429 | 71% | *RNF125, TEP1, MBTD1, GNB4, RBM43, BPNT1, EID2B, CATSPER2, DDX51, TRIM13* |  |
| 10 | 348 | 55% | *ZFP106, CCDC117, RNF13, HNRNPA0, UQCRB, PELI2, CCDC131, ARFIP1, KRAS, CNOT6* | intracellular membrane-bounded organelle (p=1.9e-11) / membrane-bounded organelle (p=2.2e-11) / intracellular part (p=1.3e-09) / intracellular organelle (p=4.4e-09) / nucleus (p=1.1e-08) / intracellular (p=2.5e-08) / nuclear part (p=1.2e-07) / nucleobase, nucleoside, nucleotide and nucleic acid metabolic process (p=3e-06) |
| 11 | 164 | 25% | *CHMP1B, CRTAP, RHEB, ARL6IP6, SBDSP, SYF2, LYPLA1, SPG21, TMEM69, ARMC10* | cytoplasmic part (p=2.1e-07) |
| 12 | 379 | 81% | *TSPAN9, ITGA2B, SEPT5, MYL9, ITGB3, SAMD14, SEC14L5, ITGB5, CDC14B, CTDSPL* | blood coagulation (p=2.9e-13) / hemostasis (p=1.1e-12) / regulation of body fluid levels (p=3.2e-12) / wound healing (p=3.1e-11) / adherens junction (p=5.5e-11) / cytoskeletal protein binding (p=1.2e-10) / plasma membrane (p=5.8e-10) / focal adhesion (p=1e-09) / cell-substrate adherens junction (p=2e-09) / plasma membrane part (p=4.1e-09) |
| 13 | 237 | 42% | *GABPA, FAM76B, TXNDC10, STAG2, AKAP11, CLINT1, JAK2, ITGAV, CBFB, ABCE1* | intracellular (p=4.2e-08) / membrane-bounded organelle (p=4.6e-07) / intracellular part (p=8.4e-07) / intracellular membrane-bounded organelle (p=8.7e-07) / nucleus (p=2e-06) / mRNA metabolic process (p=3.3e-06) / intracellular organelle (p=3.4e-06) |
| 14 | 346 | 59% | *ZBTB43, BBX, NFIC, ASXL2, PRPF40A, FICD, ANKRD13D, EIF2C2, MAP3K5, NDE1* | regulation of cellular metabolic process (p=1.3e-06) / regulation of gene expression (p=1.7e-06) / gene expression (p=2.2e-06) / regulation of metabolic process (p=2.4e-06) / RNA metabolic process (p=2.8e-06) |
| 15 | 238 | 41% | *FAU, NDUFA3, FIS1, HSPC152, UQCRQ, TCEB2, HSPC171, NDUFA13, PPP1R7, RPL36* | ribonucleoprotein complex (p=9.5e-12) / ribosome (p=2.8e-10) / cytosolic ribosome (p=9e-10) / cytoplasm (p=2.8e-09) / intracellular organelle (p=6.3e-09) / cytosolic part (p=7e-09) / intracellular part (p=8.8e-09) / intracellular organelle part (p=1e-08) / intracellular (p=1.6e-08) / structural constituent of ribosome (p=2.1e-08) |
| 16 | 125 | 17% | *HEATR1, MRCL3, NANOS1, DCBLD2, CACNA1B, DIMT1L, IL27RA, CLOCK, NDUFC1, MRPS25* |  |
| 18 | 223 | 46% | *PAK2, MEF2A, FAM21C, NR2C2, CBX5, HRB, TMOD3, MBNL3, IL6R, TPM3* |  |
| 19 | 670 | 58% | *SPRR1A, LHFPL1, MARK4, SLC27A4, UXS1, GRIN2B, PTPN5, MDFI, CHD3, ARFIP2* | transmembrane receptor activity (p=9.4e-09) / plasma membrane (p=4.1e-08) / extracellular region part (p=5e-08) / receptor activity (p=1.2e-07) / extracellular region (p=1.9e-07) / G-protein coupled receptor activity (p=5.5e-07) / signal transducer activity (p=6.3e-07) / intrinsic to membrane (p=1.1e-06) |
| 21 | 292 | 80% | *GPR56, CD247, FGFBP2, IL2RB, PRF1, EDG8, GZMA, GZMH, PYHIN1, KLRB1* | signal transducer activity (p=7.9e-14) / cellular defense response (p=2.5e-13) / immune system process (p=1.1e-12) / cell surface receptor linked signaling pathway (p=4.1e-12) / immune response (p=4.4e-12) / plasma membrane (p=5.7e-11) / receptor activity (p=6e-11) / transmembrane receptor activity (p=4.8e-08) / T cell receptor complex (p=6.4e-08) / T cell activation (p=1.2e-07) |
| 23 | 285 | 49% | *CDKN1C, PTP4A3, CKB, RHOC, IL21R, TIAM2, SCGB3A1, HES4, SFTPD, CD79B* | signal transduction (p=5.7e-14) / plasma membrane (p=1.1e-10) / immune system process (p=2.3e-10) / regulation of cellular process (p=3.5e-09) / immune response (p=7e-09) / cell surface receptor linked signaling pathway (p=8.2e-08) / intracellular signaling pathway (p=1.5e-07) / signal transducer activity (p=1.6e-07) / regulation of cell proliferation (p=1.9e-07) / transmembrane receptor activity (p=2.8e-07) |
| 24 | 68 | 12% | *SETD8, MXD3, IFI30, RNF175, GTPBP2, RN7SK, PSENEN, ALDOB, TAF10, RGS14* |  |
| 26 | 75 | 13% | *PSAP, F2R, IMAA, SALL3, RPL11, LAIR1, LILRB3, SF3A2, ARHGEF2, CLUAP1* |  |
| 27 | 200 | 31% | *SFRS14, D2HGDH, RBM33, LRCH4, TNK2, RCN3, CCDC84, CTGLF3, FAM156A, PLA2G4B* |  |
| 28 | 122 | 21% | *STRN, ATAD4, CLUAP1, VPS37D, FXC1, F2R, CLRN1, IFNA8, GPC6, ITIH5* |  |
| 29 | 142 | 23% | *PFAS, BOP1, PUS7, TTC27, TOMM40, HSPA5, PUS1, NAT10, EBNA1BP2, NOL6* | ribosome biogenesis (p=1.7e-15) / nucleolus (p=2.1e-14) / ribonucleoprotein complex biogenesis (p=8.9e-14) / nuclear lumen (p=1.9e-13) / nuclear part (p=3.4e-11) / rRNA processing (p=4.8e-10) / rRNA metabolic process (p=8.1e-10) / tRNA metabolic process (p=3.7e-09) / RNA processing (p=8.7e-09) / RNA binding (p=6.6e-08) |
| 30 | 285 | 74% | *HIST1H3F, HIST1H3H, HIST1H2AE, HIST1H2BF, HIST1H2BH, HIST2H4A, GNG8, HIST2H3C, HIST1H2BC, HIST1H2BN* | nucleosome (p<1.1e-16) / nucleosome assembly (p=1.4e-15) / protein-DNA complex (p=5.9e-15) / protein-DNA complex assembly (p=5.8e-14) / DNA packaging (p=2.1e-12) / DNA conformation change (p=6.6e-12) / chromatin assembly or disassembly (p=3e-11) / chromatin (p=3e-09) / blood coagulation (p=3.1e-06) |
| 32 | 263 | 47% | *GBP2, GBP5, GBP4, STAT1, IRF1, TAP1, GBP1, WARS, ANKRD22, VAMP5* | immune response (p=5.9e-15) / immune system process (p=5.2e-14) / antigen processing and presentation (p=8.3e-12) / defense response (p=5.4e-10) / antigen processing and presentation of peptide or polysaccharide antigen via MHC class II (p=2.2e-09) / MHC class II protein complex (p=5.2e-09) / response to stimulus (p=2.9e-08) / antigen processing and presentation of endogenous antigen (p=2.1e-07) / innate immune response (p=3.3e-07) / inflammatory response (p=4.8e-07) |
| 33 | 176 | 61% | *IFI44L, IFI44, IFIT1, MX1, IFI6, HERC5, OAS3, OAS2, MAFA, RSAD2* | response to virus (p<1.1e-16) / response to biotic stimulus (p<1.1e-16) / immune response (p=4.2e-12) / immune system process (p=1.3e-09) / response to stimulus (p=1e-07) / defense response (p=2.2e-07) / double-stranded RNA adenosine deaminase activity (p=2.8e-06) |
| 34 | 204 | 33% | *TAF15, ATP1B2, KLHL28, ZBTB40, PIAS4, BAG4, RALGDS, RAB5C, CARHSP1, PRMT1* | intracellular (p=2e-07) |
| 35 | 395 | 78% | *SLC25A39, GLRX5, ALS2CR2, WDR40A, FECH, GPR175, SNF8, GYPC, GUK1, FBXO7* | hemoglobin complex (p=3.7e-10) / oxygen transporter activity (p=8.1e-09) / oxygen binding (p=1.5e-06) / porphyrin metabolic process (p=2e-06) |
| 37 | 52 | 12% | *ZNF787, GABARAP, ITPA, MTMR14, UNC119, HMHA1, MFNG, CHMP2A, RPL13, TRAPPC6A* |  |
| 38 | 147 | 33% | *HARS2, GJA9, SRPK1, WRNIP1, R3HDM2, PIGV, MRPS25, TEAD2, GRPEL2, METAP2* |  |
| 39 | 284 | 40% | *KIR2DS4, DZIP1L, BUB1B, KCTD1, ZACN, E2F1, KIFC3, CDRT15P, EPX, SUV420H2* | intrinsic to plasma membrane (p=4.5e-07) / integral to plasma membrane (p=7.7e-07) / synaptic transmission (p=2e-06) |
| 40 | 244 | 50% | *STK19, ING3, SFRS5, MSRA, ASB8, BTK, FKBP2, BAX, RALGDS, RAB35* | protein binding (p=9.7e-07) |
| 41 | 125 | 25% | *FZD7, ERBB4, SUV39H2, DSCAM, SH2D5, FMO6P, DNA2, HOXD3, NCAN, PRAME* | transmembrane receptor activity (p=3.3e-06) |
| 42 | 264 | 44% | *ADRB2, DHRS9, PDE4B, PTPRO, UBE2J1, FAM20C, AVPI1, KYNU, GPX3, ARHGAP17* | response to wounding (p=2.7e-08) / defense response (p=4.4e-08) / membrane (p=1.3e-07) / regulation of cytokine production (p=1.3e-07) / cytokine production (p=1.5e-07) / integral to membrane (p=1.9e-07) / intrinsic to membrane (p=3e-07) / positive regulation of cytokine production (p=4.2e-07) / membrane part (p=4.9e-07) / immune response (p=5.5e-07) |
| 43 | 224 | 72% | *PACSIN1, CLEC4C, LRRC26, LEPREL1, SERPINF1, RIMS3, TLR9, TNFRSF21, FAM129C, IL28RA* | integral to membrane (p=6.8e-12) / intrinsic to membrane (p=2.7e-11) / membrane part (p=4.3e-11) / membrane (p=5.7e-08) / plasma membrane part (p=4.7e-07) / plasma membrane (p=8.9e-07) / receptor activity (p=2.1e-06) |
| 44 | 206 | 52% | *ADIPOQ, EPB41L4B, SC65, CYLC2, ZNF639, LRRN3, FMNL3, MALAT1, ATAD3C, MYF6* | neurological system process (p=7.9e-07) |
| 45 | 107 | 21% | *ADM, FLVCR2, MAP3K6, DSC2, FCGR1A, ADARB1, GAS6, BCL3, FCGR1B, FCGBP* | intrinsic to membrane (p=1e-06) / integral to membrane (p=1.4e-06) |
| 46 | 249 | 32% | *CLDN20, H2BFM, DSCR10, MGAT5B, HHATL, CTF1, COL6A2, KRT126P, SPAG5, OR2B3P* | extracellular region (p=2.4e-07) / neurological system process (p=2.5e-07) / sensory perception (p=9.7e-07) / olfactory receptor activity (p=1.5e-06) / sensory perception of chemical stimulus (p=3.5e-06) / sensory perception of smell (p=3.8e-06) |
| 47 | 181 | 41% | *INDOL1, RWDD2B, FGFBP3, S100A1, TTC7B, RNF39, HMP19, VAX2, OR2AG2, PLA2G10* | extracellular region (p=1.1e-06) |
| 48 | 311 | 72% | *TXNDC5, ABCB9, FKBP11, IGLL1, CRKRS, IGLL3, TNFRSF17, CDC20, CAMK1G, GLDC* | cell cycle process (p<1.1e-16) / cell cycle (p<1.1e-16) / M phase (p<1.1e-16) / mitotic cell cycle (p<1.1e-16) / mitosis (p<1.1e-16) / M phase of mitotic cell cycle (p<1.1e-16) / cell division (p=1.4e-13) / regulation of cell cycle (p=7e-12) / spindle (p=3.8e-11) / microtubule-based process (p=9.8e-11) |
| 51 | 135 | 55% | *HDC, SPRYD5, SLC45A3, CLC, GATA2, IL4, CPA3, MS4A3, MS4A2, CCR3* | cytokine receptor activity (p=7.2e-10) / cytokine binding (p=1.5e-08) / signal transducer activity (p=8.2e-08) / receptor activity (p=8.6e-08) / transmembrane receptor activity (p=1.2e-07) / growth factor binding (p=1.7e-06) / immune system process (p=3.3e-06) |
| 52 | 94 | 36% | *AMY1B, RPL23AP13, SIGLEC6, CNGB1, BMP8B, RFX4, ALS2CR14, STAR, AMY1A, ZNF93* |  |
| 54 | 94 | 43% | *EIF1AY, JARID1D, TMSB4Y, RPS4Y1, XIST, RPS4Y2, UTY, PRKY, ZFY, DDX3Y* |  |
| 55 | 59 | 11% | *ACOX2, EMP1, ITGB7, MT1F, LDLR, CBX7, LILRB4, E2F2, TRIM7, P2RY2* |  |
| 56 | 116 | 24% | *PADI4, CLEC5A, QPCT, PNPLA1, NR1H3, MOGAT1, COL9A2, CDK5R1, GPR177, BPI* | defense response (p=1.2e-09) / response to wounding (p=2.4e-08) / inflammatory response (p=4.4e-08) / acute inflammatory response (p=3.1e-07) / triglyceride biosynthetic process (p=8.6e-07) / extracellular region part (p=3.3e-06) |
| 58 | 261 | 48% | *ZBTB16, FKBP5, GPER, FLT3, KLF9, ADORA3, TPST1, ANKS1A, IL1R2, SMAP2* | regulation of immune system process (p=2.3e-09) / immune system process (p=2.9e-09) / regulation of developmental process (p=4.7e-08) / response to wounding (p=5.3e-08) / inflammatory response (p=9.8e-08) / humoral immune response (p=4e-07) / leukocyte activation (p=4.2e-07) / regulation of cytokine production (p=4.9e-07) / signal transducer activity (p=5.5e-07) / cell activation (p=5.7e-07) |
| 59 | 70 | 14% | *FKBP4, HSPH1, CIRBP, DNAJA4, HSPA1B, HSPB1, HSP90AA1, RBM14, UBFD1, MRPL52* | protein folding (p=2e-11) / response to unfolded protein (p=3.7e-11) / response to protein stimulus (p=4e-09) / unfolded protein binding (p=5.7e-08) / protein refolding (p=1.9e-07) / response to biotic stimulus (p=1.4e-06) |
| 61 | 86 | 15% | *SNORD16, VWA3B, SLC13A3, ZNF286A, ZNF660, STH, HUS1, ABCA4, LRRC31, PAFAH1B2* |  |
| 62 | 128 | 40% | *CRIP1, TPPP3, MYADM, EMP1, WDR49, TUBB6, RFX2, CRIP2, AGPAT4, PDE7B* |  |
| 65 | 344 | 38% | *ACTL6B, OR4K13, LEKR1, ASCL1, TMED8, NUDT11, LARP6, MT1B, CDCA8, RHBDF1* | inner ear development (p=1.9e-06) |
| 66 | 137 | 64% | *FOSB, FOS, EGR2, EGR1, ZFP36, CCL3, DUSP1, CCL3L1, CCL3L3, SGK* | response to external stimulus (p=2.8e-12) / regulation of cellular process (p=8.8e-12) / inflammatory response (p=4e-11) / response to chemical stimulus (p=1.6e-10) / response to organic substance (p=8e-10) / response to stimulus (p=2.2e-09) / behavior (p=2.2e-09) / response to wounding (p=2.9e-09) / sequence-specific DNA binding transcription factor activity (p=8.5e-09) / chemokine activity (p=1.1e-08) |
| 69 | 142 | 33% | *PALLD, FBP1, FAM124B, MRPS6, TGFA, PTGES, CD1D, GNG2, TGM2, TBC1D2* |  |
| 70 | 89 | 20% | *CLEC10A, FPR3, P2RY2, CDC42EP2, CD1C, KLHDC8B, ADAMDEC1, FCER1A, VSIG4, AVPI1* | antigen processing and presentation (p=2.5e-13) / antigen processing and presentation of peptide or polysaccharide antigen via MHC class II (p=4.4e-13) / MHC class II protein complex (p=3e-12) / plasma membrane (p=2.1e-11) / immune response (p=3e-11) / receptor activity (p=3.2e-09) / lysosome (p=3.5e-09) / immune system process (p=2e-08) / membrane (p=2.5e-08) / vacuole (p=2.5e-08) |
| 72 | 64 | 34% | *CD34, PROM1, SPINK2, MYCN, CYTL1, HOXA5, PRSSL1, SHANK3, KIT, MYH10* | defense response to fungus (p=1.3e-07) / extracellular region (p=2.5e-07) / killing of cells of another organism (p=5.9e-07) / stored secretory granule (p=9.2e-07) / response to fungus (p=2.3e-06) |
| 88 | 70 | 20% | *SASH1, GFRA2, P2RY6, CLEC10A, PID1, C1QB, C1QC, FPR3, EPB41L3, FUCA1* | membrane part (p=3.3e-06) |
| 90 | 147 | 19% | *BICD1, DNAH10, TAS2R14, MCART6, PIPOX, INPP4B, EFS, PVRL4, IFT81, CDCP1* |  |
| 92 | 31 | 8% | *ZNF329, SEDLP, ZSCAN18, ZNF671, ZNF256, ZNF274, GHRL, ZNF154, ZNF548, ZNF543* | zinc ion binding (p=2.1e-12) / transition metal ion binding (p=2e-11) / regulation of nitrogen compound metabolic process (p=6.5e-10) / regulation of cellular metabolic process (p=2.8e-09) / regulation of transcription (p=5.1e-09) / regulation of transcription, DNA-dependent (p=8.1e-09) / transcription (p=1e-08) / regulation of RNA metabolic process (p=1.2e-08) / regulation of metabolic process (p=1.4e-08) / macromolecule biosynthetic process (p=2.6e-08) |
| 93 | 135 | 34% | *DCLRE1A, SPOCD1, NEXN, AFAP1L2, GADD45A, CLEC1B, BANK1, ANGPT1, NAT8B, NFIB* |  |
| 96 | 63 | 38% | *HBA2, EPB42, ALAS2, HBB, HBM, HBD, CA1, ERAF, IFIT1L, KRT1* | hemoglobin complex (p=1.6e-16) / oxygen transporter activity (p=2.3e-14) / oxygen binding (p=5.3e-12) / defense response to fungus (p=1.3e-07) / heme binding (p=1.7e-07) / iron ion binding (p=4.6e-07) / cytosolic part (p=5.8e-07) / killing of cells of another organism (p=6.3e-07) / response to fungus (p=2.4e-06) |
| 98 | 45 | 24% | *LYZ, SHCBP1, AFMID, YEATS4, RBM44, DUSP19, HCG2P7, PCDHA11, EID2B, DDX51* |  |
| 99 | 26 | 18% | *DEFA4, ELA2, DEFA1, DEFA3, AZU1, CEACAM6, CTSG, PRTN3, CEACAM8, MPO* | response to bacterium (p=6.7e-14) / defense response to bacterium (p=2.6e-13) / extracellular region (p=1.4e-11) / killing of cells of another organism (p=3.3e-11) / response to biotic stimulus (p=1.3e-10) / defense response to fungus (p=2.2e-09) / defense response (p=1.2e-08) / killing by host of symbiont cells (p=2.5e-08) / cell killing (p=3e-08) / response to fungus (p=4e-08) |
| 102 | 14 | 11% | *RPS26L, RPS26L1, RPS26, RPS26P10, CCDC4, MADCAM1, SUOX, FBXO42, WARS2, SUSD5* |  |
| 106 | 127 | 16% | *SNORD109B, TSPAN15, HHLA1, PRX, GUCA1A, SEMA5A, PAK7, ADAMTSL5, GJB3, TAS2R5* |  |
