# Supplementary material for: Integrating Genome-Wide Genetic Variations and Monocyte Expression Data Reveals Trans-Regulated Gene Modules in Humans
Source: PLoS Genet. 2011 Dec 1;7(12):e1002367. doi: 10.1371/journal.pgen.1002367 (PMC3228821; doi:10.1371/journal.pgen.1002367)
Supplement: Table S5 — List of the 26 modules obtained by WGCNA with default parameters and enrichment of these modules in GO categories. (DOC) [file pgen.1002367.s012.doc]

**Table S5. List of the 26 modules obtained by WGCNA with default parameters and enrichment of these modules in GO categories**

| **Module** | **Number of genes in the module** | **10 most significant genes in the module (most extreme in the signature distribution)** | **Significantly enriched GO categories**  **(only the 10 most significant categories are reported when**  **more than 10 have reached Bonferroni significance)** |
| --- | --- | --- | --- |
| ME_1 | 454 | ITGB5, ESAM, CTTN, PDE5A, GP9, SMOX, GRAP2, PTCRA, PDLIM1, SH3BGRL2 | cytoskeletal protein binding (p=5.4e-10) / adherens junction (p=8.3e-10) / blood coagulation (p=1e-09) / hemostasis (p=3.2e-09) / regulation of body fluid levels (p=8.8e-09) / cell-substrate adherens junction (p=4.9e-08) / platelet activation (p=1.1e-07) / cell-substrate junction (p=1.2e-07) / wound healing (p=1.3e-07) / nucleosome (p=1.7e-07) |
| ME_2 | 750 | MED24, DPP9, TRIM28, DNM2, RFNG, DHX30, RAVER1, RIN3, MKL1, UPF1 |  |
| ME_3 | 632 | FAM49B, SBDS, PNPLA8, CD164, USP8, NXT2, NUDCD2, ECHDC1, NAT13, FAM98A | membrane-bounded organelle (p=4.9e-09) / intracellular membrane-bounded organelle (p=7e-09) / nucleus (p=1.1e-07) / nucleobase, nucleoside, nucleotide and nucleic acid metabolic process (p=1e-06) / intracellular organelle (p=1.8e-06) / macromolecule biosynthetic process (p=4.3e-06) / nitrogen compound metabolic process (p=4.3e-06) |
| ME_4 | 176 | CD247, IL2RB, TGFBR3, GPR56, PYHIN1, MATK, FGFBP2, KLRF1, PRF1, SKAP1 | signal transducer activity (p=2.7e-13) / receptor activity (p=7e-13) / immune response (p=6.5e-11) / plasma membrane (p=7.3e-11) / immune system process (p=1.1e-09) / cellular defense response (p=1.4e-09) / transmembrane receptor activity (p=1.5e-09) / cell surface receptor linked signaling pathway (p=1.5e-07) / immune response-regulating cell surface receptor signaling pathway (p=2.1e-07) / non-membrane spanning protein tyrosine kinase activity (p=3.4e-07) |
| ME_5 | 48 | PACSIN1, LRRC26, IL28RA, LEPREL1, TLR9, SERPINF1, CXCR3, ITM2C, RIMS3, FAM129C |  |
| ME_6 | 28 | TXNDC5, IGLL1, ABCB9, FKBP11, TNFRSF17, SLC25A4, IGLL3, TNFRSF13B, CD27, POU2AF1 |  |
| ME_7 | 48 | CDKN1C, IL21R, TIAM2, TSPYL3, RHOC, PTP4A3, PLAGL2, ADA, MTSS1, CD79B |  |
| ME_8 | 43 | PPIG, TTC14, RECQL, SLC25A36, ZNF654, SENP6, DIAPH2, EXOC1, PSMA4, SCARNA9 |  |
| ME_9 | 525 | UBE3A, PDS5B, HERC4, RB1CC1, FBXO11, PTBP2, ZMPSTE24, BCLAF1, BMPR2, PAPD4 |  |
| ME_10 | 290 | LARP7, SMC3, TOP2B, CD46, LSM3, SEC61G, GOLGA5, SMARCC1, DERL1, IARS2 | intracellular membrane-bounded organelle (p=9.2e-09) / membrane-bounded organelle (p=1e-08) / intracellular part (p=3.1e-08) / intracellular organelle (p=1.4e-07) / intracellular (p=6.6e-07) |
| ME_11 | 5661 | ZNF598, PI4KB, SF3B4, PIN1, ATP6V0C, GSTP1, EFHD2, MZF1, UBL7, INTS1 | extracellular region (p<1.1e-16) / olfactory receptor activity (p<1.1e-16) / transmembrane receptor activity (p<1.1e-16) / plasma membrane (p<1.1e-16) / intrinsic to membrane (p<1.1e-16) / sensory perception of chemical stimulus (p<1.1e-16) / G-protein coupled receptor activity (p=1.1e-16) / sensory perception of smell (p=3.2e-16) / integral to membrane (p=9.8e-16) / receptor activity (p=4e-15) |
| ME_12 | 131 | HAT1, RPL7, P4HA1, UBE1C, GLT8D1, GOPC, CDC5L, RPS3A, NMD3, GPR65 |  |
| ME_13 | 132 | SUCLA2, CNOT7, CYB5R4, UBE2A, UGP2, STXBP3, ACTL6A, ZC3H15, RAB5A, TMED2 |  |
| ME_14 | 99 | HERC5, EPSTI1, IFIT2, IFIT1, SERPING1, IFI35, UBE2L6, IFI6, PRIC285, XAF1 | response to biotic stimulus (p=2e-16) / response to virus (p=1.6e-14) / immune response (p=8.8e-13) / immune system process (p=1.1e-10) / response to stimulus (p=3.7e-09) / proteasome activator activity (p=1.9e-06) / hematopoietin/interferon-class (D200-domain) cytokine receptor signal transducer activity (p=4.8e-06) |
| ME_15 | 71 | LMOD3, ZNF394, TDRD1, MYO3B, XRCC2, GRIPAP1, SHROOM4, TDP1, ZNF483, HSPC268 |  |
| ME_16 | 387 | IDH3G, RALY, TWF2, APRT, ZNHIT1, ATP5D, TBCC, DPM3, NDUFB7, RPS19BP1 | ribonucleoprotein complex (p=6.1e-08) / ribosome (p=8.3e-07) |
| ME_17 | 137 | RBM12B, RHOQ, DNAJB14, COL4A3BP, GMCL1, NUFIP2, ZNF281, THOC2, TYW3, PPP2R5E |  |
| ME_18 | 20 | HDC, CLC, SLC45A3, SPRYD5, MS4A3, MS4A2, IL4, CPA3, TCN1, TRPM6 |  |
| ME_19 | 445 | SF3B5, SEC24C, ITGB4BP, EIF6, NXF1, ATP6V0D1, DDX56, UBR4, MBD1, CSNK2B |  |
| ME_20 | 358 | DTWD2, RHBDL2, FAM73A, GSTTP2, ZNF557, SLC5A8, ZADH1, PLA2G2D, ZNF669, N4BP2 |  |
| ME_21 | 508 | TIAL1, RPL34, POLR2B, PSMD10, RNF146, PLRG1, STT3A, GLUD1, RPL26, AP1G1 |  |
| ME_22 | 57 | ERAF, ALAS2, HBD, SLC25A39, ALS2CR2, HBM, TSPAN5, PFDN6, EPB42, GYPC | hemoglobin complex (p=2e-14) / oxygen transport (p=2.9e-12) / oxygen transporter activity (p=2.9e-12) / gas transport (p=1.9e-11) / oxygen binding (p=2.4e-10) / cytosolic part (p=1e-08) / heme binding (p=1.3e-06) |
| ME_23 | 206 | HNRNPA0, CEPT1, GPBP1, CMTM6, ARFIP1, CNOT6, SLC9A6, BTF3L4, PPP1R2, CSNK1G3 | nucleobase, nucleoside, nucleotide and nucleic acid metabolic process (p=1.2e-06) |
| ME_24 | 249 | BAGE5, SPATA13, ARID4B, AAK1, SSH1, TRIM38, SNRPD3, PTPRC, UGCGL1, NBPF10 |  |
| ME_25 | 763 | NGDN, PRICKLE4, NDUFS5, VPS45, STX8, PRDX1, DRG1, DBI, RPS6, UFC1 | ribosome (p=7e-08) / intracellular (p=1.2e-07) / intracellular part (p=3.3e-07) / structural constituent of ribosome (p=8.9e-07) / cytoplasm (p=2.2e-06) |
| ME_26 | 590 | SSR2, ATP5G2, PFDN1, SLC35B2, ARPC3, TTF1, NDUFA9, BANF1, SUPT5H, TBC1D14 | intracellular (p=1.8e-07) / ribonucleoprotein complex (p=1.4e-06) / intracellular part (p=1.6e-06) |
