# Supplementary material for: Integrating Genome-Wide Genetic Variations and Monocyte Expression Data Reveals Trans-Regulated Gene Modules in Humans
Source: PLoS Genet. 2011 Dec 1;7(12):e1002367. doi: 10.1371/journal.pgen.1002367 (PMC3228821; doi:10.1371/journal.pgen.1002367)
Supplement: Table S6 — List of the 71 modules obtained by WGCNA with tuned parameters and enrichment of these modules in GO categories. (DOC) [file pgen.1002367.s013.doc]

**Table S6. List of the 71 modules obtained by WGCNA with tuned parameters and enrichment of these modules in GO categories**

| **Module** | **Number of genes in the module** | **10 most significant genes in the module (most extreme in the signature distribution)** | **Significantly enriched GO categories**  **(only the 10 most significant categories are reported when**  **more than 10 have reached Bonferroni significance)** |
| --- | --- | --- | --- |
| ME_1 | 20 | HDC, CLC, GATA2, SLC45A3, SPRYD5, MS4A3, CCR3, MS4A2, IL4, TCN1 | IgE receptor activity (p=2.7e-06) |
| ME_2 | 34 | PSME2, RARRES3, SAMD4A, PSME1, OAS1, PSMB8, WARS, GNGT2, PSMB9, PLAC8 |  |
| ME_3 | 401 | ESAM, CTTN, CDC14B, GP9, PDE5A, SMOX, TSPAN9, SH3BGRL2, PTCRA, ALOX12 | blood coagulation (p=9.2e-13) / hemostasis (p=3.4e-12) / regulation of body fluid levels (p=7.4e-11) / adherens junction (p=1.6e-10) / wound healing (p=6.2e-10) / cell-substrate adherens junction (p=4.4e-09) / cytoskeletal protein binding (p=8e-09) / cell-substrate junction (p=1.5e-08) / focal adhesion (p=2.1e-08) / platelet activation (p=9.4e-08) |
| ME_4 | 657 | UBE3A, PTBP2, BCLAF1, RB1CC1, HERC4, SLK, THUMPD1, ZMPSTE24, KPNA3, PDS5B |  |
| ME_5 | 453 | RPL34, TIAL1, PSMD10, POLR2B, RNF146, GLUD1, ATP5H, STT3A, AP1G1, PLRG1 |  |
| ME_6 | 35 | APPBP2, STAG1, SLTM, ACO1, DUS4L, TOMM70A, NOC3L, ZYG11B, MIS12, PSMF1 |  |
| ME_7 | 21 | CCDC89, LY6H, PDSS2, IFNA10, ZNF655, OR11L1, CCDC140, NOL10, PITX2, DHX8 |  |
| ME_8 | 18 | FOS, FOSB, DUSP1, EGR2, ZFP36, CCL3, CCL3L3, CCL3L1, IL8, SGK | response to external stimulus (p=2.3e-12) / response to chemical stimulus (p=1e-09) / response to stimulus (p=3.4e-09) / behavior (p=4.2e-09) / response to glucocorticoid stimulus (p=3e-08) / response to corticosteroid stimulus (p=3.8e-08) / response to organic substance (p=6e-08) / response to cAMP (p=7.5e-08) / response to cytokine stimulus (p=9.9e-08) / response to extracellular stimulus (p=1.4e-07) |
| ME_9 | 139 | MTERFD1, TFB2M, KLHL20, UCHL5, SNX3, PPP2CA, EXOSC3, PNRC2, RDH14, UBL3 |  |
| ME_10 | 74 | RHOQ, RBM12B, GMCL1, UHMK1, DNAJB14, TYW3, TAOK1, PPP2R5E, FBXW7, DYRK2 |  |
| ME_11 | 71 | HNRNPA0, ARFIP1, API5, GPBP1, G3BP2, DDX21, XPO1, CSNK1G3, DDX3X, CNOT6 |  |
| ME_12 | 46 | TRIM26, COX15, SLC5A6, RPL10L, SRPR, UBAP1, TBX19, F8A1, SEMA4A, ZNF317 |  |
| ME_13 | 47 | PREX1, CCDC22, TNIP1, MRPL38, TUBGCP6, RNF166, ZNF513, HTATIP, NANS, U2AF1 |  |
| ME_14 | 68 | LMOD3, ZNF394, MYO3B, TDRD1, GRIPAP1, XRCC2, SHROOM4, TDP1, SEMA3E, ZNF483 |  |
| ME_15 | 35 | MTF2, PPARBP, THOC2, ZNF281, KRCC1, TMEM154, ZNF12, PKN2, TERF1, SRPK2 |  |
| ME_16 | 76 | GIT2, PRDX3, M6PR, MLL3, SFRS2IP, CHMP5, CTNNB1, ARMC8, RNFT1, SLC25A24 |  |
| ME_17 | 21 | SPG7, EDC4, ORAOV1, TNK2, TYK2, CCDC130, CDK10, TYSND1, QSOX2, TOP3B |  |
| ME_18 | 33 | PAIP2, MRPS30, SFT2D1, CHIC2, ITM2B, ZFAND6, ACTR10, PDCD6, NDUFB5, BRP44 |  |
| ME_19 | 72 | HAT1, P4HA1, RPL7, GLT8D1, UBE1C, LMO2, GOPC, RPS3A, NMD3, UBE4A |  |
| ME_20 | 36 | HIST1H3F, HIST1H3H, HIST1H2BH, HIST1H2BC, HIST1H2BF, HIST2H2BE, HIST1H2AE, TSC22D1, HIST2H2AA3, GNG8 | nucleosome (p=1.9e-10) / nucleosome assembly (p=1.7e-09) / protein-DNA complex assembly (p=5.8e-09) / protein-DNA complex (p=6.8e-09) / DNA packaging (p=2e-08) / chromatin assembly or disassembly (p=5.2e-08) / DNA conformation change (p=6.9e-08) / chromatin (p=1.2e-06) |
| ME_21 | 428 | FAM49B, CD164, SFRS11, SEPT7, ATP2B1, USP8, SBDS, RAB33B, R3HDM1, SPOPL | intracellular membrane-bounded organelle (p=7.7e-08) / membrane-bounded organelle (p=8.8e-08) / nucleus (p=3.4e-07) / nucleic acid binding (p=8.2e-07) / DNA ligation involved in DNA repair (p=1.2e-06) |
| ME_22 | 301 | RHBDL2, GSTTP2, DTWD2, FAM73A, ZADH1, SLC5A8, PRO1853, N4BP2, PLA2G2D, ZNF669 |  |
| ME_23 | 4902 | PTPN5, ZNF579, BBS10, ZBTB7A, CREB3, ZNF498, MRPL16, LRRC1, RAD51L1, OR7G2 | plasma membrane (p<1.1e-16) / G-protein coupled receptor activity (p<1.1e-16) / transmembrane receptor activity (p<1.1e-16) / olfactory receptor activity (p<1.1e-16) / intrinsic to membrane (p<1.1e-16) / neurological system process (p<1.1e-16) / extracellular region (p<1.1e-16) / sensory perception of chemical stimulus (p<1.1e-16) / integral to membrane (p<1.1e-16) / receptor activity (p<1.1e-16) |
| ME_24 | 86 | SF3B4, ACTN4, ZNF598, PTOV1, UNC93B1, UBL7, PNPLA6, CEP164, SBF1, PLOD3 |  |
| ME_25 | 24 | TXNIP, CLDN14, GSTCD, NME2, PPP2R2B, SCN3A, CHP, RPS24, B2M, AMY1C |  |
| ME_26 | 37 | ALAS2, HBD, SLC25A39, HBM, ALS2CR2, TSPAN5, EPB42, SELENBP1, IFIT1L, RBM38 |  |
| ME_27 | 24 | PFDN6, EEF1D, LRPAP1, PMM1, SF4, SNF8, WDR40A, ANKRD54, SSNA1, NMRAL1 |  |
| ME_28 | 93 | LRSAM1, GAK, PI4KB, E4F1, CENTA1, FAM50A, INTS1, TRABD, EPN1, PPM1F |  |
| ME_29 | 39 | ARID4A, PPIG, TTC14, RECQL, ZNF654, SENP6, SLC25A36, DIAPH2, EXOC1, PSMA4 |  |
| ME_30 | 78 | SUCLA2, STXBP3, UBE2A, CYB5R4, UGP2, ACTL6A, TMED2, PSMD6, DCK, ZC3H15 |  |
| ME_31 | 24 | TRA1P2, GPBP1L1, RPL23, FBXO38, BIN2, PAPSS1, HK2, ANP32A, ZNF226, CUL4A |  |
| ME_32 | 9 | DEFA4, ELA2, DEFA1, DEFA3, CEACAM6, CTSG, AZU1, PRTN3, MPO | defense response to fungus (p=1.7e-11) / killing of cells of another organism (p=8.2e-11) / response to fungus (p=3.2e-10) / response to bacterium (p=1.5e-08) / cell killing (p=1.7e-08) / heparin binding (p=3.5e-08) / defense response to bacterium (p=5.9e-08) / defense response (p=1.5e-07) / glycosaminoglycan binding (p=1.7e-07) / extracellular region (p=2e-07) |
| ME_33 | 40 | TCEA1, TNFAIP8, CCT6A, GNAQ, CNBP, TMF1, NCBP2, MBD2, SH3BGRL, GPD2 |  |
| ME_34 | 77 | HNRPC, TAF9, TARS, HIAT1, TANK, PPP2CB, PYGL, FAM10A4, ENO1, SAMSN1 |  |
| ME_35 | 349 | SMC3, LARP7, LSM3, TOP2B, SEC61G, SEC23IP, SKIV2L2, DDX1, NDUFB3, DERL1 | intracellular membrane-bounded organelle (p=2.6e-10) / membrane-bounded organelle (p=3e-10) / intracellular part (p=8.2e-09) / intracellular organelle (p=4.4e-08) / intracellular (p=2.8e-07) / ribonucleoprotein complex (p=9.5e-07) / nucleic acid binding (p=9.6e-07) / cellular macromolecule biosynthetic process (p=2.3e-06) |
| ME_36 | 24 | CD244, SRF, CASP9, DNAJC8, PCNT, NDST2, RP9, SNX6, IL11RA, NOL14 |  |
| ME_37 | 18 | PSMC4, IHPK2, PGAM1, TXNL2, ENSA, RFC2, RPLP0, GTF2A2, SNX20, MED27 |  |
| ME_38 | 13 | ADIPOQ, CYLC2, SC65, EPB41L4B, ZNF639, CCDC135, ATAD3C, SGTB, OR2B3P, LRRN3 |  |
| ME_39 | 41 | CDKN1C, IL21R, RHOC, PTP4A3, TIAM2, TSPYL3, CKB, PLAGL2, MTSS1, ADA |  |
| ME_40 | 112 | SIPA1, DMAP1, TJAP1, SGSM2, ZBTB48, MZF1, SNAPC4, ZNF653, EIF3B, FAM125A |  |
| ME_41 | 25 | SH2D3C, PHF19, TMC6, RRAS, SYTL1, QPRT, RGS12, CCNE1, RGL4, PRR5 |  |
| ME_42 | 69 | ATG9A, PIP5K1C, ZNF385A, RBM42, MAPK7, KCTD13, DRG2, SETD1A, SEPN1, LMF2 |  |
| ME_43 | 14 | UBXD2, VPS24, ANKRD50, PBX3, TGOLN2, ZMIZ1, IQGAP2, STK4, VKORC1L1, SSBP2 |  |
| ME_44 | 42 | MFSD3, CLK3, SNAPC2, PAFAH1B3, TUBG1, DGCR6, MAPKAPK3, ZNF342, TSSC4, KHSRP |  |
| ME_45 | 50 | CD84, TTC17, GMEB1, NMNAT1, SYK, TSR1, TAF1, SERINC3, SEPT6, GGA3 |  |
| ME_46 | 25 | TXNDC5, ABCB9, FKBP11, SLC25A4, TNFRSF17, TNFRSF13B, IGLL3, CDC20, CD27, POU2AF1 |  |
| ME_47 | 396 | SLC35B2, PFDN5, CCDC72, IGBP1, TAF12, DAP3, SSR2, EIF3I, COPZ1, MNDA | ribosome (p=1.6e-06) / ribonucleoprotein complex (p=1.8e-06) / structural constituent of ribosome (p=4.9e-06) |
| ME_48 | 15 | PUS1, TOMM40, RPP40, DPH2, NXT1, POLR3H, WDR4, SLC7A1, DDX31, PET112L |  |
| ME_49 | 43 | CD44, TMEM32, RRAGB, ANAPC4, NHS, RAB7A, IFNGR1, OSBP, NOTCH2, EXDL2 |  |
| ME_50 | 32 | UFM1, SNORD36A, SUMO2, NACAP1, IDI1, DICER1, UQCRC2, TSNAX, SNAP23, HERPUD2 |  |
| ME_51 | 341 | SF3B5, DDX56, SEC24C, ATP6V0D1, BRE, EIF6, PEF1, IQSEC1, CLN3, GPR108 |  |
| ME_52 | 414 | NGDN, DRG1, RPS6, TXN, DBI, NDUFA1, FBXL5, MRPS18C, PIGF, VPS45 |  |
| ME_53 | 77 | HERC5, EPSTI1, IFIT1, IFI6, SAMD9L, IFI44L, PRIC285, XAF1, SERPING1, UBE2L6 | response to virus (p<1.1e-16) / response to biotic stimulus (p<1.1e-16) / immune response (p=6.6e-13) / defense response (p=1.8e-11) / immune system process (p=4.9e-10) / response to stimulus (p=9.9e-09) / response to stress (p=5.9e-07) / innate immune response (p=6.8e-07) / hematopoietin/interferon-class (D200-domain) cytokine receptor signal transducer activity (p=2.3e-06) / interspecies interaction between organisms (p=2.7e-06) |
| ME_54 | 54 | AP2S1, TSPO, FAM58A, RPL18A, UBE2E2, RNASET2, CMTM7, ZNF668, MDH2, ATPAF2 |  |
| ME_55 | 160 | ARID4B, SPATA13, PTPRC, UGCGL1, AAK1, SNRPD3, TGFBRAP1, SSH1, PAPD5, TRIM38 |  |
| ME_56 | 26 | CIR, GMFG, ITGA3, GLRX, RPL37A, DC2, LCP2, PEBP1, COX6C, CYLN2 |  |
| ME_57 | 46 | PACSIN1, LRRC26, IL28RA, LEPREL1, TLR9, SERPINF1, CXCR3, FAM129C, ITM2C, RIMS3 |  |
| ME_58 | 54 | MRPS21, RP2, NCOR1, CTNNBL1, TLR6, CICE, TXNRD1, NDUFA12, RPS7, GOSR2 |  |
| ME_59 | 15 | EIF1AY, JARID1D, RPS4Y1, RPS4Y2, TMSB4Y, XIST, ZFY, PRKY, UTY, DDX3Y |  |
| ME_60 | 17 | TCEB2, RPS14, RPL36, SNRPD2, UQCRQ, TALDO1, MRPL37, MSH5, ARL16, EIF4E |  |
| ME_61 | 43 | ALDOA, RFXANK, ARPC4, TRADD, THOC4, MPDU1, CDC2L1, SIRT7, RPS9, ARPC1B |  |
| ME_62 | 51 | POMP, PSMA2, ADNP2, PPP6C, ZWILCH, NSMCE4A, SLC30A1, ACSL3, FBXO28, CEP63 |  |
| ME_63 | 163 | IL2RB, TGFBR3, GPR56, CD7, PYHIN1, MATK, FGFBP2, PRF1, NKG7, SKAP1 | signal transducer activity (p=1.5e-10) / cellular defense response (p=8.5e-10) / receptor activity (p=1.2e-09) / immune response-regulating cell surface receptor signaling pathway (p=1.3e-09) / immune response (p=1e-08) / plasma membrane (p=2.3e-08) / antigen receptor-mediated signaling pathway (p=5.4e-08) / cell surface receptor linked signaling pathway (p=6.9e-08) / immune response-activating cell surface receptor signaling pathway (p=2e-07) / non-membrane spanning protein tyrosine kinase activity (p=2.1e-07) |
| ME_64 | 26 | WBP2, GPX4, PTMS, STK40, POL3S, TBC1D13, SSBP3, NFIX, ARHGAP25, CRTC1 |  |
| ME_65 | 28 | ATG4B, SLC25A40, UBE2D3, MAEA, ARMC10, CHMP1B, SYF2, TMEM69, GALIG, NCF1 |  |
| ME_66 | 895 | DNM2, MED24, RFNG, COBRA1, IDH3G, ATP5D, DHX30, VPS72, RALY, ZNF282 |  |
| ME_67 | 48 | PLXNC1, PPTC7, RASA2, RHOT1, JARID1A, TIA1, PIK3CG, CYLD, CDKN2AIP, GNL2 |  |
| ME_68 | 55 | TBC1D23, HNRPR, TRIM52, PAPD1, SDCCAG1, FAM55C, BPTF, CCNT1, TM7SF3, VEZT |  |
| ME_69 | 448 | SUPT5H, ERGIC3, GBA, BANF1, ATP5G2, BAZ1B, PFDN1, TPI1, COMMD9, RSL1D1 | intracellular (p=1.7e-06) / ribonucleoprotein complex (p=2.2e-06) / cytoplasm (p=4.1e-06) |
| ME_70 | 15 | NDUFB6, FAU, NDUFA3, MRPL20, ARPC1A, KEAP1, NDUFB2, PCBD1, UXT, RPL29 | cellular respiration (p=1.8e-06) |
| ME_71 | 45 | PPIE, SRP14, COX17, RPL12, TUBA1C, SERF2, RAG1AP1, RILPL2, DYNLRB1, SDHA |  |
