# Supplementary material for: Integrating Genome-Wide Genetic Variations and Monocyte Expression Data Reveals Trans-Regulated Gene Modules in Humans
Source: PLoS Genet. 2011 Dec 1;7(12):e1002367. doi: 10.1371/journal.pgen.1002367 (PMC3228821; doi:10.1371/journal.pgen.1002367)
Supplement: Text S1 — Supporting information: further details of Methods, and members of the Cardiogenics Consortium. (DOC) [file pgen.1002367.s014.doc]

**Text S1**

#### The Gutenberg Health Study

#### Selection of participants for the genome-wide variability (GWV) and genome-wide expression (GWE) analyses

The Gutenberg Health Study (GHS) is designed as a population-based, prospective, observational single-center cohort study in the Rhein-Main region in western mid-Germany. The primary aim of the study is to improve the individual cardiovascular risk prediction by identifying genetic and non genetic risk factors contributing to cardiovascular diseases, with a strong emphasis on atherosclerosis.

A sample of eligible participants was randomly drawn from the registers of the local registry offices in the city of Mainz and the district of Mainz-Bingen. This sample was stratified in a ratio of 1:1 for gender and residence, and in equal numbers for decades of age. Inclusion criteria were an age between 35 and 74 years and a written consent; exclusion criteria were insufficient knowledge of the German language to understand explanations and instructions, and physical or psychic inability to participate in the examinations in the study center. The study protocol and drawing of the blood sample have been approved by the local ethics committee and by the local and federal data safety commissioners. Individuals were invited for a 5-hour baseline-examination to the study center where clinical examinations and collection of blood samples were performed. The present analysis was based on an initial sample of 3,336 subjects successively enrolled into the GHS from April 2007 to April 2008. Genomic DNA was isolated from all participants. Monocyte RNA was isolated from half of the participants recruited each day to ensure rapid sample processing and isolation of total RNA. For approximately 1,500 study participants, both DNA and RNA were available.

Peripheral blood was collected into anticoagulation tubes from each study participant during the course of examination and processed immediately. All samples were collected and processed according to SOPs. In order to ensure a minimal processing time for RNA isolation, the first tube to be taken from each participant was the tube used for isolation of total RNA.

#### Preparation of DNA

Genomic DNA was extracted from two buffy-coated EDTA blood samples (18 mL) of each participant. DNA was extracted using the method of Miller *et al* [4]. DNA yield and purity were assessed spectrophotometrically using the NanoDrop N-1000 System. DNA yield was calculated from the optical density (OD) at 260nm and DNA purity by calculating the ratio of OD260 nm and OD280 nm. For part of the samples, integrity was check on agarose gels (1 %). After quality check, DNA samples were diluted to yield 400 µg/mL.

**Preparation of RNA**

Separation of monocytes was conducted within 60 min after blood collection. 8 mL blood was collected using the Vacutainer CPT Cell Preparation Tube System (BD, Heidelberg, Germany) and 400 µL RosetteSep Monocyte Enrichment Cocktail (StemCell Technologies, Vancouver, Canada) was added immediately after blood collection. This cocktail contains antibodies directed against cell surface antigens on human hematopoietic cells (CD2, CD3, CD8, CD19, CD56, CD66b) and glycophorin A on red blood cells. Total RNA was extracted the same day using Trizol extraction and purification by silica-based columns. After separation, cells were immediately resuspended in Trizol Reagent (Invitrogen, Karlsruhe, Germany) and frozen at -20°C until isolation of RNA at the same day (maximal storage time 5 h). After thawing, samples were transferred into Phase Lock Gel Tubes (Eppendorf, Hamburg, Germany), chloroform was added and phases were separated by centrifugation. Purification of total monocytes RNA was performed using the RNeasy Mini kit (Qiagen, Hilden, Germany) according to the manufactures´ Animal Cell Spin and RNA Cleanup protocols including an additional DNase digestion step. Total RNA was eluted in RNase-free water. Yield of RNA was checked spectrophotometrically by NanoDrop N-1000 measuring the OD260 as well as the ratio OD260 and OD280. The integrity of the total RNA was assessed through analysis on an Agilent Bioanalyzer 2100 (Agilent Technologies, Boeblingen, Germany).

**Genotyping**

Genotyping was performed using the *Affymetrix* Genome-Wide Human SNP Array 6.0 and the Genome-Wide Human SNP *Nsp*I/*Sty*I 5.0 Assay kit (www.affymetrix.com). All *Affymetrix* instruments and processes were controlled by the *Affymetrix* specific software AGCC (Affymetrix Genechip Command Console 2.1). DNA samples were genotyped in batches of 96, including 94 DNA samples of the GHS, one reference genomic DNA of *Affymetrix* (Ref. 103, 50 ng/µL) and one negative control using water instead of genomic DNA. Genotyping reactions were performed as described by the *Affymetrix* user manual (www.affymetrix.com/support/downloads/manuals/genomewidesnp6_manual.pdf). Briefly, 500 ng of genomic DNA was digested using restriction enzymes *Sty*I and *Nsp*I (NEB, Frankfurt, Germany) and DNA fragments were amplified by PCR using specific oligonucleotides and the Titanium DNA Amplification kit (Clontech, Saint-Germain-en-Laye, France). After purification of PCR products by the Agencourt AMPure solid phase reversible immobilization beads technology (Beckman Coulter, Krefeld, Germany) and filter plates (Porvair, Norfolk; UK), DNA was further fragmented using DNaseI and a biotin-tag was added. Each labeled DNA was hybridized onto a microarray at 50°C and 60 rpm for 16-18 hours. After washing and specific staining steps with Streptavidin R-Phycoerythrin conjugate (Invitrogene, Karlsruhe, Germany) and a Biotin-antibody (Linaris, Wertheim, Germany), microarrays were scanned in batches of 48 samples using the Affymetrix GeneChip Scanner 3000 7G with autoloader function.

Quality of sample workflow was monitored at three steps: *i*) after PCR, size and quality of amplification products were controlled by agarose gel electrophoresis (2%), *ii*) after purification, concentration and purity of PCR products were controlled spectrophotometrically, *iii*) after DNaseI digestion, fragmentation was controlled by agarose gel electrophoresis (2.5%). After the scan process, quality of data was monitored by the Affymetrix Genotyping Console 2.1 using the feature of the QC call rate (>86%).

**Calling and quality control of genotypes**

GWV genotyping was performed in 3,306 subjects, half of them having GWE data. Genotypes were called using the *Affymetrix* Birdseed-v2 calling algorithm. Quality control was performed using GenABEL [1]. Individuals with a calling rate below 97% or a too high autosomal heterozygosity (False Discovery Rate FDR < 0.01) were excluded. Cryptic relatedness between study participants was estimated by the identity by state (IBS) statistic. In each pair showing estimated proportion of alleles IBS ≥ 0.95, the sample with the lower call rate was excluded from further analyses. Quality control was performed on 900,392 SNPs. After excluding SNPs with a minor allele frequency (MAF) ≤ 1%, or a missing rate ≥ 2% or a *P*-value for Hardy-Weinberg equilibrium ≤ 10^-4^, 675,350 SNPs were left for analysis.

**Checking for outliers and/or population stratification from GWV data**

The study population (*n* = 3,306) was examined for potential genetic heterogeneity using the first two principal components obtained by multidimensional scaling (MDS) of a matrix of pairwise IBS values between individuals. For this purpose, 18,000 SNPs were randomly selected from the 22 autosomal chromosomes and IBS was calculated using the PLINK software [2]. MDS analysis was performed on the IBS distance matrix using the isoMDS function of the MASS R package. A first run of the analysis detected 17 subjects strongly deviating from the main cluster (Figure S1). After exclusion of these 17 subjects, a second run led to the exclusion of 54 more subjects who deviated from the centre of the cluster by more than 2 SDs (Figure S2). All these subjects were subsequently identified in sample records as of non-European ancestry. A third analysis on the remaining individuals showed no apparent stratification in the data (Figure S3).

**Expression analysis**

For GWE analysis, only RNA with integrity (RIN) of ≥ 7 was used. GWE assessment was performed on 1,606 total RNA samples from monocytes using the *Illumina* HT-12 v3 BeadChip (www. Illumina.com). RNA samples were processed in batches of 96 samples. Here, 200 ng of total RNA was reverse transcribed, amplified and biotinylated using the Illumina TotalPrep-96 RNA Amplification Kit (Ambion/Applied Biosystems, Darmstadt, Germany) according the manufactures´ protocol. Steps of cDNA and cRNA purification were performed automatically using the MagMax Express96 magnetic particle processor (Applied Biosystems, Darmstadt, Germany). After amplification and purification cRNA was controlled on an Agilent Bioanalyzer 2100 (Agilent Technologies, Boeblingen, Germany) and concentration of cRNA was determined. Each sample was diluted to 140 ng/µL automatically. 700 ng of each biotinylated cRNA was hybridized to a single BeadChip at 58°C for 16-18 hours. After hybridization, BeadChips were washed and stained with 1 mg/mL Streptavidin-Cy3 conjugate (GE Healthare7Amersham Bioscience; Germany), according to the manufacture’s *Whole Genome Gene Expression with IntelliHyb Seal* Protocol. BeadChips were scanned using the *Illumina* Bead Array Reader.

**Microarray data preprocessing**

The summary probe-level data delivered by the *Illumina* scanner (mean and s.d. computed over all beads for a particular probe) was loaded in *Beadstudio*. The pre-processing done by the *Illumina* software, at the level of the scanner and by *Beadstudio* includes: correction for local background effects, removal of outlier beads, computation of average bead signal and s.e. for each probe and gene, calculation of detection *p*-values using negative controls present on the array, quantile normalization across arrays, check of outlier samples using a clustering algorithm, check of positive controls. Probes associated with the same gene identifier (as provided by manufacturer) were averaged to perform analyses at gene level. In order to reduce the variability of highly expressed genes while leaving the variability of weakly expressed genes unchanged, we applied an hyperbolic arcsinus (arcsinh) transformation to gene expression levels after all other normalizations were performed. After normalization, gene expressions were centred and standardized prior to SVD and ICA.

**Threshold for declaring a gene expressed**

Under the null hypothesis that a gene is not expressed in the cell type studied, the number of samples having a detection *P*-value < 0.05 follows a binomial distribution with parameters *n* = total number of subjects and *p* = 0.05. From this distribution, it is possible to derive an upper limit above which a gene is considered as significantly expressed in the study population. The threshold for significance was corrected for the number of genes (*m* = 37,804, *P* = 1.32 x 10^-6^). With this corrected threshold, a gene was declared expressed when the proportion of samples with a detection *P*-value < 0.05 was > 7.8%.

**Checking for outliers and/or stratification from GWE data**

Similarly to GWV analysis, the first two components obtained by MDS were used to detect outliers in GWE data. The distance between any 2 individuals was computed as 1 minus the absolute correlation between the 2 arrays. Ten individuals whose data were located more than 4 s.d. away from the center of the main cluster were excluded from further analyses (Figure S4).

**Singular value decomposition (SVD) analysis prior to ICA**

The screeplot of SVD eigenvalues was used to determine the minimum number of factors required to explain non-random variation (Figure S5). We developed a method based on random permutations adapted from [3]. The distribution of eigenvalues under H_0_ (absence of correlation structure) was obtained by applying SVD to 10 random matrices obtained by permuting individuals independently for each gene expression. Data were reconstructed from the first *k* factors of the SVD, *k* being the optimal number determined.

#### The Cardiogenics Study

#### Selection of participants for the genome-wide variability (GWV) and genome-wide expression (GWE) analyses in the Work Package 5

The present study included 459 patients with coronary artery disease (CAD) or myocardial infarction (MI) and 458 healthy individuals of European descent recruited in five centers within the Cardiogenics consortium ([http://www.cardiogenics.eu](http://www.cardiogenics.eu/)). Healthy individuals were recruited in Cambridge (UK). CAD/MI patients were recruited in Leicester (*n*=161), Lubeck (*n*=102), Regensburg (*n*=122) and Paris (*n*=74). The study was approved by the Institutional Ethical Committee of each participating center.

**RNA and DNA extraction**

Monocyte isolation, macrophage culture and RNA extraction were performed separately in each center according to standardized procedures. All RNA samples were subsequently sent to one center (Paris) for amplification, gene expression profiling and bioinformatics analysis.

Blood samples (30 ml) from fasting subjects were collected into EDTA and monocytes were isolated with CD14 micro beads (Miltenyi) according to the manufacturer’s instructions Monocyte purity was measured as the percentage of CD14+ve cells analyzed by flow cytometry. Half of the isolated cell preparation was immediately used for RNA extraction and the remaining cells were cultured for 7 days at 37°C in Macrophage-SFM medium (Invitrogen, Cat # 12065-074) supplemented with glutamine (1mM; Invitrogen) and recombinant human M-CSF (R&D Systems), the latter taken from the same batch. At day 4 the medium was replaced and at day 7 the cells were harvested. Isolated monocytes and 7 day macrophages were lysed in Trizol and RNA was extracted in chloroform and ethanol, washed in RNeasy columns and incubated with DNase I before extracting in RNase-free water. RNA was quantified by the Nanodrop® method before transfer to Paris on dry ice.
In addition, EDTA antigoagulated venous blood samples were collected from all participants. Genomic DNA was extracted from peripheral blood monocytes by standard procedures (Qiagen) and sent to the Sanger Institute for genotyping.

**Whole-genome transcriptional profiling**

Gene expression profiling was performed using the *Illumina* Human Ref-8 Sentrix Bead Chip arrays (*Illumina* Inc., San Diego, CA) containing 24,516 probes corresponding to 18,311 distinct genes and 21,793 Ref Seq annotated transcripts. mRNA was amplified and labelled using the *Illumina* Total Prep RNA Amplification Kit (Ambion, Inc., Austin, TX). After hybridization, array images were scanned using the *Illumina* BeadArray Reader and probe intensities were extracted using the Gene expression module (version 3.3.8) of the *Illumina* *BeadStudio* software (version 3.1.30). Raw intensities were processed in R statistical environment using the Lumi and beadarray packages as described in Maouche et al. (to be submitted). All array outliers were excluded and only arrays with high concordance in terms of gene expression measures (pairwise Spearman correlation coefficients within each cell type > 0.85) were included in the analyses. After data quality control, 849 monocyte RNA samples were available for statistical analyses.

**Genome-wide genotyping, samples and SNPs filtering**

Genome-wide genotyping was carried out using two *Illumina* arrays, the Sentrix Human Custom 1.2M array (1,115,839 SNPs and 80,128 CNVs) and the Human 610 Quad Custom array (594,398 SNPs and 66,049 CNVs). After sample filtering based on sample call rate, ancestry, duplications and genetic relatedness, 802 samples were kept for eQTL analyses. For SNP filtering, SNPs were removed if (i) they had a per-SNP call rate lower than 95% in cases or controls on the two arrays, and/or (ii) a (MAF) ≤ 1% in cases or in controls and/or (iii) a MAF in controls generated using the *Illumina* 1.2 M or in those generated on the 670k array and/or (iv) a significant deviation from Hardy-Weinberg equilibrium in controls (*P* < 10^-5^). Furthermore, all SNPs on the Y and mitochondrial chromosomes as well as CNV markers were excluded from statistical analysis. After quality control, 522,603 SNPs were used for eQTL analyses.

**eQTL analyses**

eQTL analyses were carried out in 758 individuals. Analyses of variance with adjustment on age, gender and center were performed in R statistical environment.

**Members of the Cardiogenics Consortium not included in the manuscript**

Tony Attwood^1^, Stephanie Belz^2^, Peter Braund^3^, Jason Cooper^4^, Abi Crisp-Hihn^1^, Jeanette Erdmann^2^, Nicola Foad^1^, Jay Gracey^3^, Emma Gray^5^, Stefanie Gulde^2^, Rhian Gwilliams^5^, Susanne Heimerl^6^, Jennifer Jolley^1^, Unni Krishnan^3^, Heather Lloyd-Jones^1^, Ingrid Lugauer^6^, Per Lundmark^7^, Jasbir S Moore^3^, David Muir^1^, Elizabeth Murray^1^, Chris P Nelson^3^, Jessica Neudert^8^, David Niblett^5^, Karen O'Leary^1^, Helen Pollard^3^, Angela Rankin^1^, Catherine M Rice^5^, Hendrik Sager^2^, Jennifer Sambrook^1^, Gerd Schmitz^9^, Michael Scholz^8^, Laura Schroeder^2^ , Ann-Christine Syvannen^7^, Chris Wallace^4^

*^1^Department of Haematology, University of Cambridge, Long Road, Cambridge, CB2 2PT, UK and National Health Service Blood and Transplant, Cambridge Centre, Long Road, Cambridge, CB2 2PT, UK; ^2^Medizinische Klinik 2, Universität zu Lübeck, Lübeck Germany; ^3^Department of Cardiovascular Sciences, University of Leicester, Glenfield Hospital, Groby Road, Leicester, LE3 9QP, UK;  ^4^Juvenile Diabetes Research Foundation/Wellcome Trust Diabetes and Inflammation Laboratory, Department of Medical Genetics, Cambridge Institute for Medical Research, University of Cambridge, Wellcome Trust/MRC Building, Cambridge, CB2 0XY, UK; ^5^The Wellcome Trust Sanger Institute, Wellcome Trust Genome Campus, Hinxton, Cambridge CB10 1SA, UK; ^6^Klinik und Poliklinik für Innere Medizin II, Universität Regensburg, Germany; ^7^Molecular Medicine, Department of Medical Sciences, Uppsala University, Uppsala, Sweden;* *^8^Trium, Analysis Online GmbH, Hohenlindenerstr. 1, 81677, München, Germany; ^9^Institut für Klinische Chemie und Laboratoriumsmedizin, Universität, Regensburg, D-93053 Regensburg, Germany*

**References**

1. Aulchenko YS, Ripke S, Isaacs A ,van Duijn CM (2007) GenABEL: an R library for genome-wide association analysis. Bioinformatics 23:1294-1296.

2. Purcell S, Neale B, Todd-Brown K, Thomas L, Ferreira MA, et al. (2007) PLINK: a tool set for whole-genome association and population-based linkage analyses. Am J Hum Genet 81:559-575.

3. Horn JL (1965) A Rationale and Test for the Number of Factors in Factor Analysis. Psychometrika 30:179-185.

4. Miller SA, Dykes DD, Polesky HF. A simple salting out procedure for extracting DNA from human nucleated cells. *Nucleic Acids Res*. 1988;16:1215.
